# Mapping variation in dual use risk assessments of synthetic biology projects

**DOI:** 10.3389/fbioe.2025.1620678

**Published:** 2025-08-14

**Authors:** Daniel Greene, Tessa Alexanian, Megan J. Palmer

**Affiliations:** ^1^ Center for International Security and Cooperation, Stanford University, Stanford, CA, United States; ^2^ iGEM Foundation, Paris, France; ^3^ Department of Bioengineering, Stanford University, Stanford, CA, United States

**Keywords:** dual use research of concern (DURC), biosafety, biosecurity, synthetic biology, biotechnology, risk assessment

## Abstract

**Introduction:**

When a life science project is identified as potential dual use research of concern (DURC), United States government policy and biorisk management professionals recommend conducting a risk assessment of the project and using its results to choose strategies to manage any associated risks. However, there is little empirical research on how real-world projects score on DURC assessments, the extent to which reviewers agree or disagree about risks for a given project, or how risk judgments map to recommended risk management strategies. By studying the process of DURC risk assessment, it may be possible to develop methods that are more consistent, accurate, and cost-effective.

**Methods:**

Using a modified version of the framework in the Companion Guide to the United States Government Policies for Oversight of Life Sciences Dual Use Research of Concern, we elicited detailed reviews from 18 experienced DURC reviewers and 49 synthetic biology students of the risks, benefits, and recommended risk management strategies for four real-world synthetic biology projects.

**Results:**

We found significant variation among experts, as well as between experts and students, in both perceived DURC risk and recommended risk management strategies. For some projects, expert risk assessments spanned 4 out of 5 possible ratings. We found substantial disagreement between participants about the appropriate actions to take to manage the DURC risks of each project.

**Discussion:**

The observed variation in participants’ judgments suggests that decisions for similar projects may vary significantly across institutions, exposing the public to inconsistent standards of risk management. We provide several research-based suggestions to reduce reviewer disagreement and manage risk more efficiently when reviewers disagree.

## 1 Introduction

United States Government policy has defined “dual use research of concern” (DURC) as “life sciences research that, based on current understanding, can be reasonably anticipated to provide knowledge, information, products, or technologies that could be misapplied to do harm with no, or only minor, modification to pose a significant threat with potential consequences to public health and safety, agricultural crops and other plants, animals, the environment, materiel, or national security” (Office of Science and Technology Policy, 2024a, p. 8). If funders, research performers, government regulators, publishers, or other stakeholders are concerned that a research project has DURC potential, they can perform a risk assessment and identify strategies to manage any perceived risk (Office of Science and Technology Policy, 2024b; National Academies of Sciences, Engineering, and Medicine (2018); Greene et al. (2023); Casagrande (2016); Tucker (2012); Vennis et al. (2021). Current federal policy requires certain projects to undergo DURC risk assessment and encourages researchers to proactively consider risk for others (Office of Science and Technology Policy, 2024a). This policy and its predecessor (Office of Science and Technology Policy, 2014c) have faced mixed reactions from researchers, some of whom argue that they overlook research of concern, disincentivize valuable research, and take undue time (Evans et al., 2021; Bowman et al., 2020).

Data on reviewer judgment are critical to studying and improving the DURC risk management process. As the current policy states, “It is essential to have a common understanding of and consistent and effective implementation for research oversight across all institutions that support and conduct life sciences research” (Office of Science and Technology Policy, 2024b, p. 7). In practice, DURC is typically managed by small groups of expert reviewers. Research institutions subject to the United States DURC policy are required to evaluate research projects in groups of five or more, known as Institutional Review Entities or IREs, and case studies of biorisk management practices suggest that small-group discussion is also common in non-United States research institutions that assess DURC (Greene et al., 2023). Little is known about how real-world projects score on DURC risk assessment frameworks, the extent to which reviewers agree or disagree about the risks of a single project, or how their judgments of risk map to recommended risk management strategies (Bowman et al., 2020). For example, it could be the case that reviewer groups with a certain key combination of skill-sets tend to produce final opinions that they judge as particularly high-quality, or that reviewers tend to reach their final opinions after a certain amount of time and that providing additional time is unnecessary. This study is an initial attempt to provide data to inform the design of DURC risk management.

In this study, we elicited a set of DURC risk assessments of four synthetic biology projects from two different reviewer groups: experienced DURC project reviewers (referred to here as “experts”), and synthetic biology students, who typically receive little education on dual-use issues (Minehata et al., 2013; National Research Council, 2011; Vinke et al., 2022). We asked participants for their estimates of project risks and their recommendations for strategies to manage those risks. The overall aim of our study is to collect data on reviewers’ judgments of potential DURC projects to suggest improvements to the DURC risk management process and to illuminate areas for future research. We focus primarily on two topics: the extent of expert reviewers’ agreement or disagreement about potential DURC and the differences between expert and novice opinions.

Our primary research goal was to assess the degree to which individual reviewers agreed or disagreed about how to manage the potential risks of a common set of potential DURC projects. We expected that reviewers would vary in their project assessments, given that deep disagreements exist regarding the correct conceptual frameworks to use for risk assessment, appropriate thresholds of risk tolerance, relevant ethical values to consider, and which stakeholders to include (National Academies of Sciences Engineering and Medicine, 2017; Selgelid, 2016; Dryzek et al., 2020). Although small-group discussion is the norm for assessing potential DURC, we collected data from individual reviewers because it would have been difficult given the scope of our study for us to coordinate large numbers of reviewer groups and compensate them for their time. However, as we explain later, our data can be used to make inferences about the opinions of groups; if individual reviewers often disagree with one another then small groups selected from a population of those individuals may not represent the entire range of opinions in the population. This is problematic because it would imply that IREs at different institutions apply different standards to evaluate similar projects, even though they could impose similar risks onto the public. Later, we review strategies for promoting reviewer agreement and for intelligently managing DURC risks in the absence of agreement.

Our second research goal was to compare the opinions of relative experts and novices in dual-use risk management. As the life sciences grow in scale and the potential for DURC increases, society may need a larger supply of skilled DURC reviewers (Bowman et al., 2020). We examine how experts and novices rate a common set of projects and how they view the risks of the field of synthetic biology as a whole. By better understanding expert-novice differences, we hope to identify key elements of knowledge and skill that can be used to train and identify skilled project reviewers more efficiently in the future. It may also be possible to identify areas in which expert reviewers are not needed because novices provide comparable judgments. Increasing the supply of reviewers and streamlining the review process also reduces the time and financial cost of DURC risk assessment, making it more likely that organizations will choose to perform it.

## 2 Methods

We integrated our research into the International Genetically Engineered Machine (iGEM) competition, an annual event in which teams of junior synthetic biologists from around the world (typically college undergraduates) spend the summer developing and executing their own synthetic biology projects (Mitchell et al., 2011). The competition has been running since 2003 and maintains extensive documentation of past projects. iGEM also has a robust safety and security program and a large staff of volunteer expert reviewers (Millett and Alexanian, 2021). Thus, as a research venue, iGEM provided populations of novices, experts, and past projects that could be used as a basis for comparative evaluation.

We surveyed 49 recent iGEM participants (hereafter “students”) and 18 biorisk experts (“experts”) between July and November 2021. Students voluntarily signed up to participate based on advertisements sent to recent iGEM participants, and experts signed up based on advertisements to a pool of past iGEM project reviewers and DURC risk assessment experts. Each participant filled out a short survey about their background, completed DURC risk assessments for up to four real past iGEM projects, and recommended possible risk management strategies for each project (See the Supplementary Material for more information.) Our relatively small sample sizes provide 80% power to detect between-group t-test differences of d=.78 or greater. To translate to our primary outcomes of interest, this value corresponds to a between-group difference of about 0.9 scale points on the 1-5 risk scale used in our question about project DURC risk, and a difference of about 1.8 in the average number of risk mitigation strategies that a participant could endorse for a given project.

Because the study posed minimal risk to participants, it was approved as exempt from review by the Stanford University Institutional Review Board (IRB). All participants completed consent forms before participating that explained the nature and purpose of the study.

### 2.1 Identifying synthetic biology projects that pose realistic DURC concerns

We drew from past iGEM projects to ensure that we were working with realistic examples of DURC risks in synthetic biology. We ran a pilot survey with a separate pool of experts to select projects that posed sufficient DURC concern to merit review, but that were varied enough that we expected them to be assessed differently from one another (see Supplementary Material). Under current United States policy none of these projects would be required to undergo a formal review of their DURC potential, as they do not involve any listed agents, toxins, or experimental outcomes of concern. However, current policy explicitly notes that some DURC may not involve the listed agents, toxins, or outcomes, and encourages PIs and research institutions to “remain vigilant” and manage risks appropriately (Office of Science and Technology Policy, 2024b, p. 12). Our findings are therefore most relevant to potential DURC that evades current list-based policy frameworks (Millett et al., 2023). A description of each of the four projects used in this study is provided in Table 1.

**TABLE 1:** iGEM projects with DURC potential reviewed in this study.

Project (original iGEM team)	Intended benefits	Potential risks	Risk management strategy
Insect Vectors: Delivering bacteriophages to crops using insect vectors (Wageningen_UR 2019)	Prevent bacterial infections of crops by spreading a protective bacteriophage through insect vectors	Insights into adhesion of bacteriophages to insect vectors might be repurposed for transferring harmful phages	Team developed a safe-by-design approach after discussing dual use concerns with local biosafety experts and consulted with iGEM on a responsible communication plan
Non-canonical AAs: Developing translational machinery for non-canonical amino acids (Bielefeld-CeBiTec 2017)	Expanding the space of possible protein designs by using non-canonical amino acids and unnatural base pairs	Alternative codons might allow evasion of DNA synthesis screening, while non-canonical amino acids might allow design of novel protein toxins	Team consulted with experts, prepared a report on safety aspects of the expanded genetic code, and wrote a paper on biocontainment strategies in iGEM (Whitford et al., 2018)
Immune Escape: Enabling immune escape for a fungal cockroach pathogen (SZU-China 2018)	Enhance the efficacy of a fungus used as a pest control solution for cockroaches, which can carry pathogens and parasites	Methods used to enhance the lethality of the fungal pathogen (engineered adhesion proteins and immune evasion) might be repurposed to enhance other pathogens	Team engineered a kill-switch to limit environmental spread, but did not address dual use; iGEM considers this project a near miss and added new policies on gain-of-function projects in 2019
Psilocybin: Engineering *E. coli* to manufacture psilocybin (Sydney_Australia 2019)	Reduce the cost of researching psilocybin as a treatment for mental health conditions	Inexpensive production of a substance that is illegal in many parts of the world might lead to individual harms from exposure, crime and psychoactive substances stolen from the lab	Team obtained local regulatory approval to work with psilocybin and outlined safety approaches such as working in elevated containment, limiting concentrations produced, and managing lab access

For each of the four projects we created a 4-8 page packet of background material for reviewers, similar to what project leads might provide in a real scenario (see Supplementary Material). The packets were condensed and anonymized versions of team’s responses to the iGEM safety form, a self-assessment of risk that every iGEM team is required to complete for their project midway through its development. We did not include information about iGEM’s original assessments of project risks or their risk management efforts.

### 2.2 Designing a DURC risk assessment form

We derived a DURC risk assessment form largely from the United States Government’s 2014 DURC policy companion guide for academic institutions (National Institutes of Health, 2014). We chose this guide because it offers a detailed set of questions for assessing the risks and benefits of a project, provides guidance for developing a risk management plan, and is likely widely used by IREs. Other DURC risk assessment forms are focused more narrowly on risks or are intended to assess biotechnologies more broadly rather than individual projects (National Academies of Sciences Engineering and Medicine, 2018; Tucker, 2012; Vennis et al., 2021).

Three years after we collected our data in 2021, the United States Government updated the 2014 DURC policy (Office of Science and Technology Policy, 2014c), integrating it with an updated framework for managing risks of enhanced potential pandemic pathogens (ePPPs) and issuing a new companion guide (Office of Science and Technology Policy, 2024a). However, the methods and results of this study remain applicable to DURC management today. Both the old and new policies would not require IREs to review the projects in this study because they do not involve the required biological agents, toxins, or experimental outcomes, but both policies recognize the potential for such projects to be DURC. Both policies also require IREs to evaluate projects for DURC and have functionally similar guidelines for forming and operating an IRE. Finally, the companion guides for both policies provide very similar guidance on how to assess risks and benefits and select risk mitigation strategies, directly reusing sections of text and covering similar subheadings (e.g., type of misuse, ease of misuse, and potential consequences) (National Institutes of Health, 2014; Office of Science and Technology Policy, 2024a).

In our study, the risk assessment process for a single project first involved participants reading the background material about the project and answering questions about their initial reactions (Students were also asked to provide an initial judgment of the overall DURC risk of the project; see Results for more information.) All participants then completed a series of reflection questions adapted from the DURC policy companion guide about the nature, magnitude, and likelihood of potential harms and benefits of the project, including both open-ended and Likert-scale questions. They were also carefully guided through the United States Government’s definition of DURC. After completing the reflection questions, they provided summative 5-point ratings of the overall perceived DURC risk and overall perceived benefits of the project.

Finally, we asked participants whether they would recommend applying any of eight separate risk management strategies to the project (Table 2). Again, these strategies were derived from those suggested in the DURC policy companion guide and adapted to the iGEM context (e.g., “more monitoring from iGEM leadership” rather than “more monitoring from the United States Government”). For each strategy, participants could select one of four options: “not necessary”, “doesn’t seem necessary but the team could consider it”, “seriously consider”, or “the team should not continue without this strategy”. To simplify analysis, we labeled the former two options as “non-endorsement” of the strategy and the latter two as “endorsement”. All surveys and assessment forms are included in the Supplementary Material.

**TABLE 2:** Risk management strategies for DURC risk in iGEM projects.

Strategy	Description provided to reviewers
More training	Should the team complete additional training that is relevant to the specific risks of the project? This might include: special training for handling certain strains or chemicals, or seeking expert advice about the risks in the project
More monitoring	Should the team monitor their work more closely and report more frequently to iGEM? This might include: providing ongoing regular reports to iGEM, or identifying milestones that require the project to be reviewed again by iGEM before proceeding
Improve biosafety	Should the team implement additional biosafety measures? This might include: using additional physical containment (e.g., a biosafety cabinet, or BSC) or working at a higher biosafety level (BSL); using additional PPE; or completing an internal biosafety risk assessment
Improve biosecurity	Should the team implement additional biosecurity measures? This might include: increased security of the physical lab space or materials (e.g., adding access controls); increased security for computers or digital information; or more careful review of the people involved with the project
Study countermeasures	Should the team evaluate the efficacy of existing countermeasures against the organisms or parts produced by the project? If no effective countermeasures (medical, detection, or otherwise) exist, the team would consult with iGEM about how to proceed
Manage communications	Should the team craft a plan for responsible communication of the research results and review it with iGEM before proceeding? This might include: redacting specific information in light of security concerns; highlighting the biosafety and biosecurity measures used during the course of the project; or emphasizing the public health or other beneficial uses of the project
Modify project	Should the team modify its experimental design or methodology? This might include: utilizing an attenuated strain or employing molecular containment measures that limit a strain’s ability to proliferate outside the lab or within different hosts
Stop project	Should the team stop the project? It may be the case that no other options can sufficiently address the risks of the project. In this case, the team should stop the project

## 3 Results

### 3.1 Participation summary

49 students and 18 experts completed ratings of one or more projects. Details on participant recruitment, data cleaning, and our statistical approach are provided in the Supplementary Material. All participants could choose to assess up to four projects, and students and experts each assessed approximately the same number of projects (student mean = 2.35 projects, expert mean = 2.00 projects, t=.97 , df=32.05 , p=.34 ). Each individual project was assessed by 8–11 experts and 27–30 students. Participants were equally likely to complete a risk assessment of any of the four projects ( $\chi^{2}=3.16$ , df=3 , p=.37 ).

### 3.2 Participant backgrounds and beliefs about synthetic biology

Before assessing any projects, participants answered a set of questions about their background experience and beliefs and attitudes related to synthetic biology and biological risk. Their responses helped us to contextualize participants’ perspectives, characterize differences in perspective between experts and students, and confirm commonsense notions of “expertise” in terms of training and familiarity with biological risk.

As expected, experts reported more education, experience, and familiarity with synthetic biology and biological risk than did students. 16 of 18 experts held a Masters’ degree or greater, compared to 10 of 49 students. Experts were also more likely than students to report having multiple years of experience with wet-lab work (11 of 18 experts, 16 of 49 students) or with “broader aspects of synthetic biology (e.g., human practices, sociology of science, risk analysis)” (10 of 18 experts, 7 of 49 students), though the groups had comparable dry-lab experience (3 of 18 experts and 7 of 65 students reporting “years” of experience). Finally, on a 1–5 scale, experts reported significantly more prior familiarity with the concept of DURC (expert mean = 4.29, student mean = 2.28, t=9.03 , df=44.08 , p<.001 ).

**FIGURE 1:**
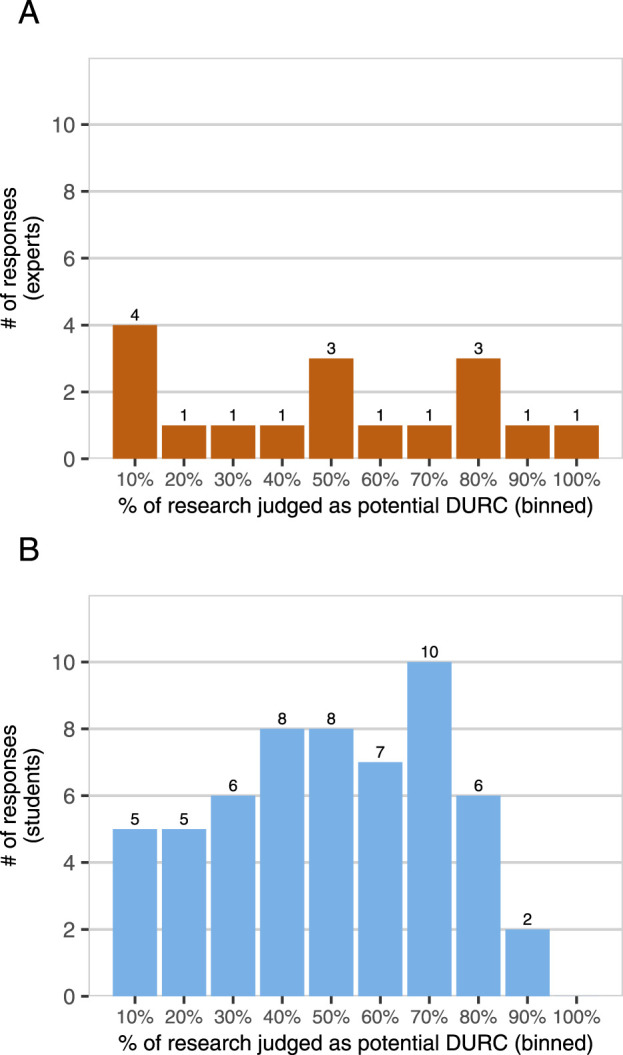
*DURC potential in synthetic biology?* Experts [Panel **(A)**, n = 17] and students [Panel **(B)**, n = 57] were asked “In your opinion, what percentage of synthetic biology research being done today has the potential to be DURC?” Participants were also provided with the United States government definition of DURC for reference and asked for a value between 0% and 100%. Responses are binned to the nearest higher multiple of 10, e.g., 61%–70%.

**FIGURE 2:**
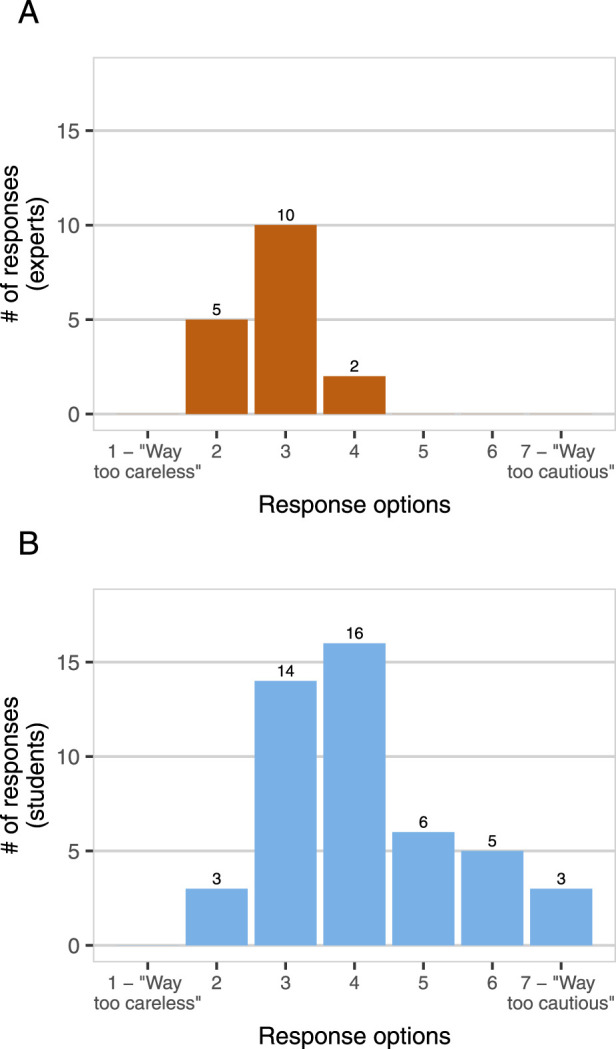
*Too cautious or too careless?* Experts [Panel **(A)**, n = 17] and students [Panel **(B)**, n = 47] were asked “Do you think that synthetic biology researchers are generally too cautious about the potential risks of their work, or too careless about those risks, or somewhere in between?” Response options were provided on a 7-point Likert scale from “Way too careless” to “Way too cautious”, with a midpoint at “Neither too careless nor too cautious”.

Experts and students also differed in their reported perspectives on DURC risks (Figure 1) and risk management (Figure 2) in the field of synthetic biology. We asked participants whether synthetic biology researchers are generally “too cautious” or “too careless” about the risks of their work. No experts reported that synthetic biology researchers are “too cautious”, but 29% of students did (1–7 scale, expert mean = 2.82, student mean = 4.11, t=−5.23 , df=56.40 , p<.001 ). In addition, experts and students did not significantly differ in their mean estimates of the percentage of current synthetic biology research that poses DURC potential (expert mean = 45.82, student mean = 47.74, t=−0.24 , df=21.61 , p<.81 ), but experts had far more variance in their estimates, which ranged from 3% to 100% (expert $\sigma^{2}=930.28$ , student $\sigma^{2}=544.96$ , permutation test p<.001 ). Taken together, these results suggest that while the experts in our sample recommend care and caution in synthetic biology, they differ substantially from one another in their perceptions of the scale of potential DURC.

After reading each project description, participants rated their understanding of the project and their familiarity with its topic and its methods on 5-point scales. There were no statistically significant differences between expert and student groups on any of these dimensions. After providing summative 5-point ratings of the overall risk and benefits of the project (discussed below), participants also rated their confidence in their own ratings on a 5-point scale. On all of these measures there were also no statistically significant differences between experts and students in omnibus tests or on a per-project basis (all p>.1 ). This suggests that while experts and students had different levels of background education, attitudes, and familiarity with DURC, they did not significantly differ in their self-assessed familiarity with or confidence in assessments of the specific projects presented in this study.

### 3.3 Variation in perceptions of DURC risk

After rating the magnitude and likelihood of potential harm for each project, participants were asked to rate its overall DURC risk on a 5-point scale, following the United States Government definition of DURC: *“Overall, combining your judgments of the potential harms of misuse with the likelihood of misuse … How much risk do you think that the knowledge, information, technology, or products from this project pose to public health and safety, agricultural crops and other plants, animals, the environment, materiel, or national or global security?”*

**FIGURE 3:**
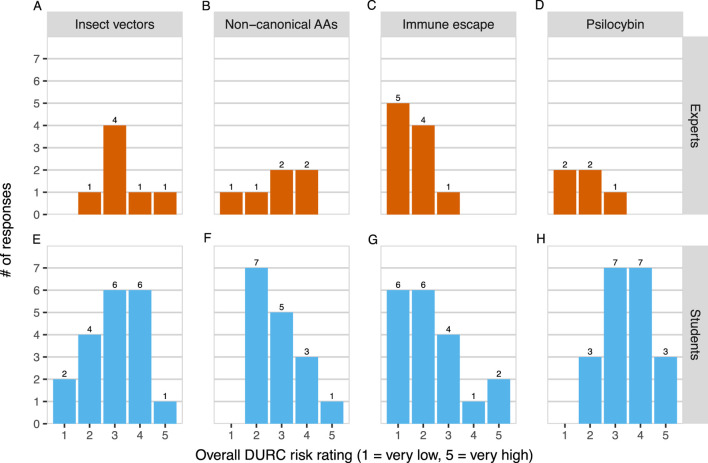
Experts [Panels **(A–D)**] and students [Panels **(E–H)**] rated the DURC risks of four research projects on a 1-5 scale from “very low” to “very high”.

We found that risk ratings for most projects varied widely, with two projects spanning 4 points of the 5-point rating scale even within the small expert group (Figure 3). In other words, expert participants disagreed substantially about the perceived risk of several projects. As a result, the mean risk ratings of three out of the four projects were also statistically indistinguishable from one another ( F(2,70)=0.474 , p=.625 ). The only standout was the “Immune escape” project, whose mean risk rating was marginally or significantly lower than that of the other three projects in pairwise comparisons (with “Insect vectors”: p=.005 , 95% C.I. = [−1.78, −0.23]; with “Non-canonical AAs”: p=.06 , 95% C.I. = [−1.60, 0.01]; with “Psilocybin”: p=.002 , 95% C.I. = [−1.87, −0.31]).

We also examined expert-student differences in risk ratings. As shown in Figure 1, experts and students held a similar average perception of the percentage of synthetic biology research with DURC potential. However, experts rated our selection of projects as about 0.6 scale points *less* risky on average than did students on a 1-5 scale (expert mean = 2.32, student mean = 2.93, t=−2.72 , df=44 , p=.0097 ). Examining individual projects once again, we found that differences were largely due to the psilocybin project (expert mean = 1.80, student mean = 3.50, t=−3.95 , df=6.82 , p=.006 ) and the immune-escape project, though the latter difference was not statistically significant (expert mean = 1.60, student mean = 2.32, t=−1.93 , df=26.93 , p=.06 ).

We also hypothesized that experts would have less variance than students in their risk ratings because they might draw from a common body of knowledge and experience to arrive at more similar conclusions to each other. However, we did not find evidence that experts’ project risk ratings have any less variance than students’ ratings using an omnibus permutation test across all projects (p=.51) or subsetting the data to individual projects (all p>.1 ).

### 3.4 Perceived benefits and enthusiasm about projects are uncorrelated with perceived DURC risk

We hypothesized that someone who was familiar with or particularly excited about a project’s topic might also be likely to view it as being less risky. To test this hypothesis, we asked participants how excited they felt about each project, how familiar they were with its topic and methods, the magnitude of the project’s potential benefits, the likelihood that the benefits would be realized, and the overall expected benefits of the project. None of these factors were significantly correlated with a project’s overall DURC risk rating (all p>.1 ).

We also compared experts and students’ ratings of the overall expected benefits of the projects. On average, experts judged the four projects in this study as being 0.53 points less beneficial than did students on a 5-point scale, a marginally significant result (expert mean = 2.50, student mean = 3.03, t=2.38 , $\chi^{2}=3.11$ , p=0.078 ).

### 3.5 Variation in recommended risk management strategies

**FIGURE 4:**
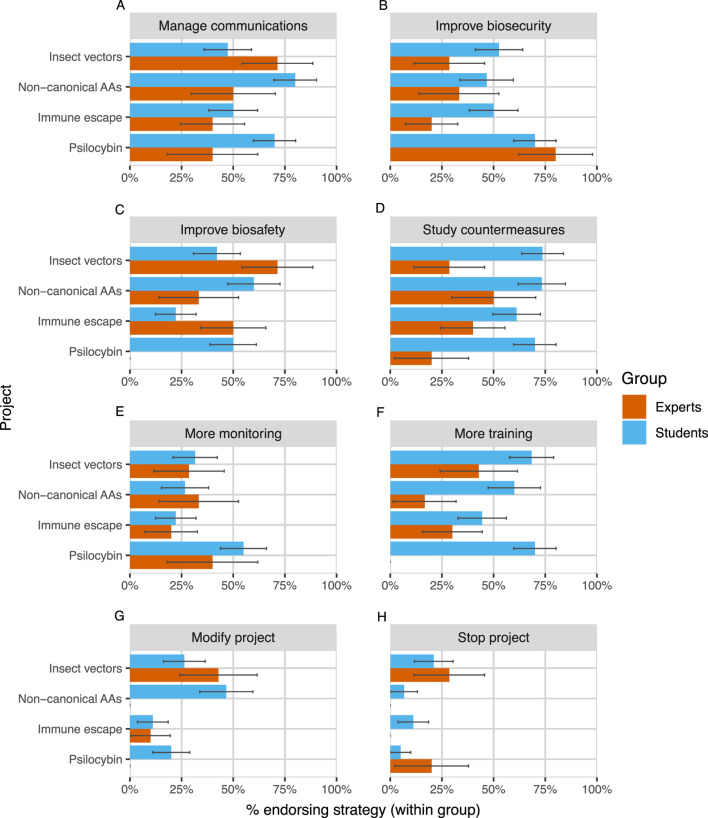
Expert and student endorsement of eight risk management strategies [Panels **(A–H)**] across projects. Strategies are presented in descending order of average endorsement among experts. Error bars represent ± one standard error, estimated using the normal approximation of a binomial proportion confidence interval (Wikipedia Contributors, 2024).

We found substantial disagreement between expert participants about the appropriate actions to take to manage the DURC risks of research projects. On average, two randomly selected experts who evaluated the same project only agreed on whether to endorse about five out of eight possible strategies (60%). Experts typically did not endorse modifying or stopping projects and were therefore much more likely to agree about those strategies (81%) than about other, less disruptive strategies (53%; see Figure 4). Still, 2 out of 8 experts endorsed stopping “Insect vectors” and 1 out of 8 endorsed stopping “Psilocybin”. Expert agreement also varied depending on the project under consideration, from 50% of strategies for “Insect vectors” to 70% for “Psilocybin”.

We also observed differences between students and experts in the number and type of strategies that they endorsed (Figure 4). Despite more frequently reporting that synthetic biologists are too cautious in general, students endorsed a larger number of strategies per project on average than did experts (expert mean = 2.43, student mean = 3.61, $\chi^{2}=4.60$ , p=0.032 ). In particular, across every project a greater percentage of students than experts endorsed the strategies of “More training” and “Study countermeasures.” Students and experts also appeared to disagree strongly about the “Modify project” strategy for the non-canonical amino acids project, and about the “More training” and “Improve biosafety” strategies for the psilocybin project.

Overall, the four projects did not vary in their average number of endorsed risk management strategies ( F(3,96)=1.52 , p=.21 ), though an individual’s rating of a project’s DURC risk was highly correlated with the number of strategies that they endorsed ( r=.49 , p<.001 ). Across all projects, some risk management strategies were also far more likely to be endorsed than others ( F(7,792)=14.21 , p<.001 ). “Study countermeasures” and “Manage communications” were the most popular (60 and 58 endorsements respectively out of 100 total evaluations), and “Modify project” and “Stop project” were the least popular (22 and 11 endorsements respectively). The most and least popular strategies also varied from project to project, suggesting that reviewers were sensitive to the specific features of each project. For example, both experts and students were relatively likely to endorse “Improve biosecurity” for the psilocybin project (presumably to prevent the theft of illegal drugs), but not for other projects.

### 3.6 The process of risk assessment does not significantly shift risk perceptions among students

Student participants were asked to rate the DURC risk of each project twice - once immediately after reading about the project and a second time after completing the reflection questions based on the United States Government’s DURC policy companion guide. We hypothesized that the process of completing the reflection questions might cause students to shift their initial risk estimates. For example, the questions might help students to realize their own degree of uncertainty about the potential benefits and harms of a project, a phenomenon known in general as the illusion of explanatory depth (Fernbach et al., 2013; Rozenblit and Keil, 2002). However, in paired t-tests, we found that students’ post-reflection risk ratings were statistically indistinguishable from their pre-reflection ratings across all four projects ( t=−0.21 , df=73 , p=0.83 ) and for each individual project (all p>.1 ).

## 4 Discussion

Managing DURC requires assessing the risks of projects and then choosing actions to manage those risks. However, there is very little empirical research on DURC risk assessment that compares the opinions of multiple reviewers across a fixed set of projects, and it is unclear how much reviewers agree about how to manage the risks of any given project. To address this lack of research, we compared the assessments of experienced biorisk evaluators and recent iGEM participants on a common set of synthetic biology projects. In order to make the assessment process more realistic, we adapted an existing process from the United States Government’s DURC evaluation companion guide and applied it to four real past iGEM projects. We also collected a set of common risk management approaches (Table 2) and asked reviewers about which strategies they would recommend.

We found substantial variance in risk ratings and management strategy recommendations across all four projects, even within the expert group. In some cases, expert risk assessments spanned 4 out of 5 possible ratings, and experts were divided roughly 3-to-1 about whether to stop one project entirely. Expert opinions about the overall percentage of synthetic biology research with DURC potential ranged from near 0% to near 100%. As a group, experts also had no less variance in their project ratings than did students.

### 4.1 Implications of expert disagreement

The observation that experts disagree about DURC risk management is concerning because it suggests that the public is being exposed to inconsistent standards of risk tolerance regarding potential DURC. A central premise of risk management is that decisions should be aligned with the risk tolerance of all stakeholders exposed to the risk. For example, air safety regulators should take the risk tolerance of passengers into account when deciding how much to spend on mitigating risks of plane crashes (Aven, 2015). By definition, DURC projects have the potential for broad effects, both positive and negative, that could affect large fractions of society no matter where they are carried out. Therefore, DURC management decisions should arguably be aligned with the risk tolerance of the public.^1^

But DURC reviewers disagree substantially about the risks and appropriate management strategies for the projects in our study, and about the fraction of synthetic biology research with DURC potential. This suggests that reviewers at different research institutions likely apply different standards of risk tolerance to the projects that they see, and these standards cannot all be aligned with the public’s risk tolerance at the same time. Therefore, some reviewers are likely making decisions about potential DURC that do not align with the public’s risk tolerance.

### 4.2 Generalizing from individuals to groups

Diversity of perspective is a key reason why groups are commonly used in DURC review. Ideologically diverse groups are more likely to arrive at a position that is both higher quality and more consistent with the independent opinions of other such groups (McBrayer, 2024). Ideally, IREs should be composed of individuals who will collectively raise all perspectives on a given project that are worthy of serious consideration.

However, our findings suggest that IREs may sometimes lack perspectives that are common enough to plausibly deserve serious consideration, but also rare enough to be left out purely by chance. Our reasoning is as follows.

For two out of the four projects we studied (“Non-canonical AAs” and “Immune escape”), experts were in full agreement to continue the project, and we will not consider them further. For the other two projects, 2 out of 8 experts (25%) endorsed stopping “Insect vectors” and 1 out of 8 (12.5%) endorsed stopping “Psilocybin”. While we lack ground truth about whether stopping these projects is worthy of serious consideration, it seems plausible, given that they reflect more than 10% of expert opinion in each case and that the costs of a mistake may be high.

If we treat the opinions in this sample as representative of a larger population of experts, how likely is it that a randomly selected group of five reviewers (the minimum size required for an IRE by the United States Government) (Office of Science and Technology Policy, 2024b) contains no one who would wish to stop each project? For “Insect vectors”, the estimate is ${\left(1-2/8\right)}^{5}=23.7\%$ . For “Psilocybin”, the probability is ${\left(1-1/8\right)}^{5}=51.3\%$ . In both cases, there is a non-trivial probability that a five-person IRE would fail to include anyone who believes that the project should be stopped. Furthermore, if an IRE lacks an initial dissenting perspective, group polarization (whereby a group that is already in agreement further cements its agreement through discussion) would make it less likely that any individual, much less an entire group, would change its position (Sunstein, 1999) (This phenomenon is consistent with anecdotal reports from institutions that their IREs approve nearly all projects (Bowman et al., 2020)).

### 4.3 Limitations of this study

Our study may overestimate the variance in experts’ risk ratings compared to real-world DURC risk assessments for at least three reasons. First, as we have noted above, the projects in this study were assessed by individuals rather than groups, but previous expert recommendations and federal guidelines posit that DURC risk assessment should involve multiple reviewers with diverse backgrounds in discussion with one another. Groups tend to outperform individuals on a wide range of decisions, including forecasting decisions (Kerr and Tindale, 2004; Tindale and Winget, 2019). We expect that group assessments of DURC risk would have less variance than individual assessments, on average, because groups can collectively access and integrate a wider range of perspectives and evidence. For example, groups might be more likely than individuals to know about the capabilities of malicious actors, the base rates of biotechnology project success, or technical methods of dispersing pathogens.

Second, we sought out example iGEM projects that had raised concern in the past, but the rules of the iGEM competition exclude a great deal of work that could be clearly judged as high-risk, such as work with pathogens that would require BSL-3 or BSL-4 precautions. As a result, the projects in this study were likely to be neither obviously safe nor obviously dangerous. Our findings may therefore be most helpful for gauging the level of current consensus about particularly high-variance “edge cases” that can fall outside of typical guidance frameworks and are most in need of expert review (Evans and Palmer, 2018). Future empirical work should attempt to include clear low- or high-risk projects as benchmarks for comparison. Our personal opinion is that the large majority of synthetic biology research projects have little to no DURC potential, but the findings of this study suggest that a substantial fraction of iGEM students and expert reviewers may disagree. More work is essential to clarify and resolve this disagreement.

Third, our study may overestimate the real-world variance in risk ratings because it did not give reviewers the opportunity to ask research proposers for more information about their projects, as is recommended by the United States Government companion guide for DURC assessment (Office of Science and Technology Policy, 2024b). Several reviewers in our study wrote in open responses that they found it difficult to assess the risks of some projects because they felt that they lacked sufficient information.

On the other hand, our study also simplified reviewers’ opinions about risk management strategies in a way that could have reduced variance in opinion. We converted opinions from a four-point scale of “not necessary”, “doesn’t seem necessary but the team could consider it”, “seriously consider”, or “the team should not continue without this strategy” into a binary variable of endorsement vs non-endorsement. While this simplification allows for a much more straightforward analysis, it may mask important differences of opinion. The most extreme scale points more clearly represent the reviewers’ opinions about whether to use a given strategy, while the middle two points are less clear. Future studies, or reanalyses of the data in this study, could reveal important disagreements about risk management.

In addition, geographical factors could have contributed to differences in opinion between experts and students. All 18 experts were from the United States, Canada, or Europe, while the students were from all inhabited continents except Australia. It is possible that the students in this study were less capable than the experts at understanding the English-language written materials, though both groups expressed similar levels of understanding of each project. It is also possible that the students held a different or wider range of cultural expectations regarding risk management than experts, though the experts and students had similar levels of variance in their final judgments of project risk.

### 4.4 Suggestions for improving DURC risk management

What should be done if independent assessments of potential DURC, performed with groups and given the opportunity for researcher feedback, could still plausibly come to radically different conclusions about how to manage risks? We offer several suggestions below, divided into three categories: improving group deliberation, aligning standards for risk tolerance, and reducing the costs of risk management.

#### 4.4.1 Improving group deliberation

One source of reviewer disagreement about potential DURC arises from differences in knowledge about the nature, magnitude, and likelihood of harms that might arise. To reduce disagreement, DURC risk assessment could be studied and re-engineered to guide reviewers toward informed consensus via good-faith deliberation that is grounded in a well-curated set of relevant background knowledge.

First, risk assessment forms could supply relevant crucial considerations identified from past assessments as background information for future reviewers. It could be possible to identify and inform reviewers about crucial considerations that commonly arise with certain topics. Studying crucial considerations could help to clarify the nature of DURC risk assessment expertise, to democratize access to this expertise, and to make assessment faster and potentially more accurate.

Here are several examples of possible crucial considerations that could be supplied to reviewers.

• *Likelihood of project success.* On average, experts rated both the risks and benefits of projects lower than novices did, and experts endorsed fewer risk management strategies. One possible hypothesis for this pattern is that experts may expect projects to be less likely to succeed in general because they have more experience seeing promising research ideas fail in execution. Experts were significantly less excited than students about projects on average (2.57 vs. 3.38 on a 5-point scale, $\chi^{2}=6.33$ , p=0.012 ). Future research could test this hypothesis more directly by measuring expert and novice assumptions about the likelihood that a given project is successfully realized. If a difference between experts and novices proves to be reliable, perhaps reviewers could be provided with data about the base rates of project success to help them make more expert-like judgments. However, it should be noted that a project does not always need to be successful to create risks. Some dual-use risks may be created merely through the dissemination of certain ideas or knowledge conveyed by a project, regardless of whether the project achieved its goal.

• *Costs of risk management.* We found that students were more generally more likely than experts to endorse all risk management strategies (particularly “More training” and “Study countermeasures”). One possible explanation for this finding is that the experts were more aware of the costs and delays that could be caused by these strategies. Future work could include more information about the costs of risk management strategies, or it could ask reviewers more directly about perceived costs.

• *Definitions of terms.* Experts and students in our study disagreed significantly about the risks of the psilocybin project. One possible reason for this finding is that the two groups held different implicit views about whether illicit drug production should be considered in scope as a “dual-use” issue. A simple clarification in the instructions could address this and similar issues.

• *Background on actors who might misuse DURC.* DURC reviews sometimes involve considering the pool of actors who are willing and/or able to deliberately misuse life science research to cause harm. It may be valuable to supply DURC reviewers with some basic level of publicly available background information about such actors. For example, data might include the approximate numbers of people who have expertise in certain research techniques or estimates of the size of major international terrorist organizations.

Second, future work could identify areas of agreement and remove them from assessment. Our study found some points of potential consensus or near-consensus among experts. Future work could follow up on these points and refine them into rules and standards that could be removed from the risk management process entirely, saving reviewers time. For example, biosecurity measures were recommended more than 80% of the time for the “Psilocybin” project in this study. This suggests a possible rule of thumb: potential DURC involving psychoactive compounds should follow higher biosecurity standards by default (perhaps unless the researchers involved specifically apply for an exception).

Third, future work could improve DURC risk assessment rubrics to make it easier to understand sources of disagreement. In this study we adapted a rubric from United States Government guidelines that focuses on the likelihood, magnitude, and overall risk of harm. This rubric’s expected value framework is more informative than a single risk rating, but it does not convey reviewers’ personal theories of what exactly could be harmed and how. Rubrics should require reviewers to make their theories explicit in order to clarify disagreements about the underlying causes of risks and to explain why they recommend some risk management strategies over others. DURC risk assessment rubrics should therefore include not only global scores of likelihood, magnitude, and overall risk, but also explanations of specific potential harms (such as damage to public health or agricultural crops) and their imagined mechanisms (such as an accidental lab escape).

Finally, future work could adopt domain-general best practices for group deliberation. Group decision-making is one of the first topics ever studied by psychologists, and many well-established findings could be applied to improve the quality of DURC review (Triplett, 1898; Kerr and Tindale, 2004; Tindale and Winget, 2019). For example, once assembled to make a decision about a project, members should first reflect in private and then each share their initial thoughts in full before starting discussion. This ensures that groups are working with all available information, reduces the chance of groups anchoring on the first ideas that are mentioned, and makes it harder for confident or higher-status members to dominate discussion (Brodbeck et al., 2007; Littlepage et al., 1997; Whyte and Sebenius, 1997). IREs should consider making this practice standard procedure for reviewing life science research projects, and future research could examine the current deliberation practices employed by Institutional Biosafety Committees (IBCs) and measure the effects of research-backed deliberation practices on IBC judgments.

#### 4.4.2 Aligning reviewers’ standards of risk tolerance

Reviewers of potential DURC not only hold different knowledge about potential risks, they hold different values and worldviews that can contribute to disagreement (Dake, 1991). Values and worldviews inescapably shape how people conceptualize risk and decide how or whether it should be managed. While a full treatment of the role of values and worldviews in risk management is outside the scope of this article, one way that they are expressed is through risk tolerance, or the degree of risk that an individual is willing to accept from some activity (McBrayer, 2024). In judging a research project as DURC, reviewers must implicitly or explicitly decide that the risks of the project are below some threshold of acceptability, but their personal thresholds may not be aligned with one another or with the thresholds of members of the public that would be exposed to risks from DURC.

One potentially promising strategy for setting a defensible level of risk tolerance comes from United States Nuclear Regulatory Commission policy for managing the risks of nuclear power plant accidents (Nuclear Regulatory Commission, 2024). The Commission adopts a very conservative lower bound of risk tolerance that they assume virtually all members of the public would accept, and then implements any risk management strategies that are needed to bring risk below this threshold for all citizens. In principle, the federal government could apply a similar approach with DURC research. Regulators could model the risks of harm to the public from potential DURC, use a standardized conservative risk-tolerance estimate for the public, and then implement whatever risk management was needed to meet this standard.

Unfortunately, it is arguably far more difficult to model the risks involved with DURC than the risks of nuclear accidents. While nuclear accidents involve a series of events that are relatively contained in time and space and typically involve mechanical failures and human error, DURC risks involve the unfolding of emerging technologies across the globe, over ambiguous timescales, and potentially involving the strategic decisions of malicious actors. Still, a common threshold for DURC risk tolerance could help reviewers to clarify their disagreements and move debates forward.

#### 4.4.3 Lowering the costs of risk management when disagreement is unavoidable

Even with the techniques described above, it may sometimes be difficult or impossible to reach consensus about a project’s risks. But if the cost of managing risks is low, then consensus about the size of those risks is less necessary to make a clear decision. Researchers and risk management professionals could reduce the cost of DURC risk management by researching fundamental improvements to biosafety methods, developing alternative research methodologies that produce similar forms of knowledge with less risk of misuse, building infrastructure for responsible data- and code-sharing, more widely sharing existing risk management practices, or simply intervening earlier in a project’s lifecycle (Greene et al., 2023; Dettmann et al., 2022; Ritterson et al., 2022; Rozell, 2015; Sandbrink and Koblentz, 2022; Smith and Sandbrink, 2022).

It might even be worth exploring the potential for less-experienced reviewers to perform DURC risk assessment at lower cost but with little sacrifice in quality. In our study, we did not observe a clear consensus among experts about project risks or appropriate risk management strategies, and students and experts differed in some cases but not others. It would be premature to casually dismiss the role for experts in DURC risk assessment without more research. But if, upon further study, experts and non-experts ultimately provide similarly murky judgments of risk, non-experts could help to assess DURC in contexts or at scales where it is difficult to access expertise or where simple triage judgments are sufficient for useful action.

DURC risks from life sciences research are increasing as technology matures and is disseminated, and our results suggest that risk management experts disagree substantially about risks and appropriate risk management strategies. Society would benefit from a more mature field of DURC risk management that is based on a foundation of empirical data. This article illustrates the potential for such data to clarify DURC management decisions and to inspire improvements to the risk management process.

## Data Availability

The datasets presented in this study can be found in online repositories. The names of the repository/repositories and accession number(s) can be found below: Zenodo Dataset, record id: 15238461, doi: 10.5281/zenodo.15238461, url: https://zenodo.org/records/15238461.

## Footnotes

1 While the concept of a single level of risk tolerance for the public might seem incoherent, governments frequently make regulatory decisions that necessarily assume a certain degree of risk tolerance from the public, such as mandating insurance requirements and requiring safety features on automobiles, just as they assume a monetary value for lives saved through investments in public safety. In both cases, the need to balance a nonzero probability of harm and a cost of risk management implies a balancing point that governments choose and impose on their citizens (Aven, 2015; Duprex et al., 2015).
