# Supplementary material for: Mapping variation in dual use risk assessments of synthetic biology projects
Source: Front Bioeng Biotechnol. 2025 Aug 14;13:1620678. doi: 10.3389/fbioe.2025.1620678 (PMC12392780; doi:10.3389/fbioe.2025.1620678)
Supplement: Supplementary file 1 [file DataSheet1.pdf]

Supplementary Material - Mapping variation in DURC risk assessments of synthetic biology projects

Daniel Greene, Tessa Alexanian, and Megan Joan Palmer

1 Selection of past iGEM projects with DURC potential **2**
1.1 Choosing candidate projects 2
1.2 Selecting projects for the study 5
2 Final project descriptions provided to reviewers **7**
2.1 Project #1: "Insect Vectors" 7
2.2 Project #2: "Non-canonical AAs" 14
2.3 Project #3: "Immune Escape" 18
2.4 Project #4: "Psilocybin" 23
3 Supplemental methods: recruitment, participation, data cleaning, and statistics **31**
4 Background survey **34**
5 DURC risk assessment form **37**

1 Selection of past iGEM projects with DURC potential

The four past iGEM projects with DURC potential included in the study were chosen based on a set of stakeholder interviews and a survey.

1.1 Choosing candidate projects

We interviewed several long-time iGEM stakeholders about projects that they remembered as raising DURC concerns. Based on the stakeholder interviews, we identified 8 candidate projects. For each project, experts were presented with a description of the project (derived from the team's iGEM Safety Form) and a possible dual-use concern. The project descriptions are given in the table below in the order they were presented to experts.

Table S1: *Past iGEM projects provided to experts*

Project	Anonymized description	Possible dual use concern
Insect Vectors: Delivering bacteriophages to crops using insect vectors (Wageningen_UR 2019)	Our project aims to develop a biological system for the control of the grapevine infecting plant pathogen: Xyella fastidiosa. This will be executed by producing modified bacteriophages from a modified carrier host that will produce the phages upon coming into contact with the quorum sensing molecule of X. fastidiosa. We will equip the bacteriophages with the same spreading potential as X. fastidiosa, which makes it able to use the same insect vectors. This genetically engineered bacteriophage will also encode for specific peptides that are released upon phage-mediated lysis and trigger an immune response in plants. On top of this, we want to develop a detection device for the presence of X. fastidiosa in the insects, enabling real-time detection and consequently the ability to highly specifically administrate the phage therapy.	Technology for insect delivery of bacteriophages might be repurposed to deliver plant pathogens.
Non-canonical AAs: Developing translational machinery for non-canonical amino acids (Bielefeld-CeBiTec 2017)	We aim at establishing a toolkit for the translational incorporation of non-canonical amino acids in E. coli. As an example for the successful incorporation of non-canonical amino acids we will develop a rapid test for prions and a new chromatography method for a mild elution of proteins. Moreover, we want to develop an E. coli strain which can retain unnatural	A toolkit for use of non-canonical amino acids might allow bad actors to evade mitigation measures such as DNA screening that relies on natural base pairs.

	base-pairs between isoG and isoC on expression vectors.	
Immune Escape: Enabling immune escape for a fungal cockroach pathogen (SZU-China 2018)	Our project is going to engineer the Metarhizium anisopliae (a kind of fungi that can kill cockroaches) by enhancing the ability of attachment and infection, helping them to evade immune cell recognition. And finally, suicide when they expose to outside the cockroaches.	Evasion of immune system recognition for a fungal insect pathogen could constitute gain-of-function research.
Exosome delivery: platform delivery technology using exosomes capable of crossing the blood-brain barrier for CNS RNA therapy (XJTLU China 2018)	Our human embryonic kidney HEK293 cells will produce exosomes transporting specific mRNA into target neuronal cells. We will increase the expression of related several proteins to accelerate the generation of exosomes, and mRNA in the producer cells is specifically packaged through the interaction between the membrane protein CD63 linked to L7Ae and a specific sequence in the mRNA. These exosomes permeate the blood-brain barrier and target neuronal cells via RVG on the exosomal membrane. The mRNAs are released into the neurons with the help of the channel protein, connexin.	Exosome delivery mechanism could be used as the basis for a platform to deliver harmful mRNA to neurons.
Psilocybin: Engineering *E. coli* to manufacture psilocybin (Sydney_Australia 2019)	We are attempting to move the biosynthetic pathway for psilocybin from the mushroom Psilocybe cubensis into Escherichia coli. We will be cloning four metabolic enzymes required for the conversion of tryptophan to tryptamine to 4-hydroxytryptamine to nor-baeocystin to baeocystin to psilocybin. Psilocybin is a psychedelic drug that has shown efficacy in the treatment of affective and anxiety disorders such as depression and post-traumatic stress disorder. A reliable, scalable, and efficient method of psilocybin production is necessary to enable further clinical research and provide a cost-effective source of the pharmaceutical, if it becomes more widely used as a medicine.	Engineered E. coli could be used to synthesize illegal drugs.
Viral engineering: modular expression of virus-like particles intended for vaccine	The goal is to create an easy and fast way for everyone to modify Virus-like particles (VLPs), which can be used as vaccines or drug-delivery systems, by engineering an E.	Faster development of VLPs might enable development of intentionally harmful VLPs.

development and targeted drug delivery (TU_Darmstadt 2019)	coli strain to express those VLPS and modify them by transforming the desired cargo/ exterior protein into the strain. In particular we decided to use the exterior of the Salmonella typhimurium P22 bacteriophage. VLPs only consist of the viral proteins but do not contain any viral DNA or RNA, consequently they are not infectious. To assemble the P22 procapsid (PC) consisting of so called coat proteins (CP) we need a scaffolding protein (SP) which ensures the composition (self-assembly) of the CPs. This mechanism is navigated by the C- Terminus of the scaffold protein which interacts with the coat protein. After in vivo synthesis of the VLP, it can be modified on the outside using an enzyme called sortase A7M (Ca2+-independent version of the Staphylococcus aureus enzyme sortase A. This transpeptidase binds the decoration of interest covalently to the VLP. For doing so we are going to mark our coat protein with a LPETG-tag via genetic engineering which now can be found by the sortase. The nanoparticles cannot only be modified to present something on the outside, but can also contain cargo on the inside. This could be achieved by designing a fusion protein consisting of scaffold and cargo. As the result of our work we want to design a bacterial strain that expresses the VLP. After assembly the antigen or other cargo will be attached by the sortase. This bacterial strain should be easy to modify so that the antigen or cargo on the interior and/or the exterior surfaces can be changed quickly depending on the desired utilization.	
Gene drive: construction of a gene drive in yeast (Minnesota 2016)	We plan to create two haplotype strains of yeast. One haplotype will be the gene drive. The other haplotype will have the recovery drive. Both drives will be driven by CRISPR-Cas9. We will then mate the haplotype containing the gene drive with the wild type of the other haplotype to verify that the gene drive does work. The other haplotype with the recovery drive will be mated with the wild type of the other	Knowledge from yeast gene drive development could be used to create a gene drive that causes environmental harm.

	haplotype and in another experiment with the gene drive haplotype. Tetrad pulling will be done to verify if the recovery drive worked.	
Living contraceptives: characterization of spermicidal peptides intended to be produced by vaginal microflora (Montpellier 2018)	Our project is to create a new contraception method. We are aiming at engineering Lactobacillus jensenii (bacteria from the vaginal flora) and Bacillus subtilis (as a model organism for Gram positive bacteria) in order to make them produce spermicidal peptides and antibodies. The peptides are Nisin (BBa_K1365000), Subtilosin (BBa_K1697000), LL-37 (BBa_K1162006), Magainin, Lacticin ; the antibodies are antisperm antibodies produced by the human body that we will synthesize.	Microbial contraceptive technology might be misused by bad actors interested in non-voluntary contraception.

1.2 Selecting projects for the study

To select the final projects, we then surveyed 9 experts recruited from the iGEM Safety and Security committee about dual-use concerns in those projects. Experts were asked to answer the following questions about each project:

1. Which of the following areas could plausibly be harmed by the misuse of this project? Please check all that apply.
    a. Public health and safety
    b. Agricultural crops and other plants
    c. Animals
    d. The environment
    e. Materiel
    f. National or global security
2. Overall, how much risk do you think that the knowledge, information, technology, or products from this project pose to the areas that you checked above (if any)?
    a. Very low risk
    b. Moderately low risk
    c. Moderate risk
    d. Moderately high risk
    e. Very high risk
3. How much risk is created by the physical products of the project?
    a. None at all
    b. A little bit
    c. A moderate amount

    d. A large amount
    e. A great deal
4. How much risk is created by the knowledge and information produced by the project?
    a. None at all
    b. A little bit
    c. A moderate amount
    d. A large amount
    e. A great deal
5. Overall, how large or small are the expected benefits of this project?
    a. Very small
    b. Moderately small
    c. Moderate
    d. Moderately large
    e. Very large

We averaged ratings across all 9 experts for each project and compared the projects in overall risk and benefits, variance of risk and benefits, and ratio of risk created by the physical products versus the knowledge and information produced.

We chose the following four projects to capture a diverse range of responses:

- *Insect vectors*: Low variance in overall risk and benefits, novel type of risk (not only affecting human health)
- *Non-canonical amino acids*: Lowest average overall risk score
- *Immune escape*: Highest risk score for both physical products and information
- *Psilocybin*: High variance in perceived risk and benefits, relatively low information risk and relatively high physical risk

2 Final project descriptions provided to reviewers

For each of the four projects we condensed and anonymized team responses to the iGEM safety form, a self-assessment of risk that every iGEM team is required to complete for their project midway through its development.

2.1 Project #1: “Insect Vectors”

Describe the goal of your project: what is your engineered organism supposed to do? Please include specific technical details and names of important parts.

Our project aims to develop a biological system for the control of the grapevine infecting plant pathogen: *Xyella fastidiosa*. This will be executed by producing modified bacteriophages from a modified carrier host that will produce the phages upon coming into contact with the quorum sensing molecule of *X. fastidiosa*. We will equip the bacteriophages with the same spreading potential as *X. fastidiosa*, which makes it able to use the same insect vectors. This genetically engineered bacteriophage will also encode for specific peptides that are released upon phage-mediated lysis and trigger an immune response in plants. On top of this, we want to develop a detection device for the presence of *X. fastidiosa* in the insects, enabling real-time detection and consequently the ability to highly specifically administrate the phage therapy.

Which whole organisms, including viruses and cell lines, are you planning to use or using in your project?

Philaenus spumarius
Xanthomonas campestris pv. campestris (LMG568)
Vitis vinifera
Brassica oleracea var. capitata
S. cerevisiae CEN.PK2-1D
E. coli K-12 MG1655
E. coli BL21.DE3
E. coli BL21-Rosetta 2
E. coli DH5Alpha
E. Coli DH10Beta
E. coli JS200
E. coli JW3367
E. Coli LE392
Bacteriophage Lambda
Bacteriophage T7

What risks could these organisms pose to you or your colleagues in the laboratory, or to your community or the environment if they escape the lab?

These organisms are of no harm considering human health. Considering the environment, *X. campestris* is a plant pathogen which is already present in our country.

X. campestris is a BSL-2/risk group 2 organism which can do no harm to humans, but it is a plant pathogen. Proper handling instructions for working with this organism will be done when plant infection assays will be executed. This will be done and supervised by a specialized lab at our institution where they previously have done infection assays with this particular organism.

What organisms are you using as chasses in your project?

●	Escherichia coli (give names of all strains you are using)<br>- E. coli BL21.DE3, E. coli BL21-Rosetta 2, E. Coli DH10Beta, E. coli DH10BetaTM, E. coli dh5alpha, E. coli JS200, E. coli JW3367, E. Coli LE392, E. coli K-12
●	Yeast (Saccharomyces cerevisiae)
●	Lactobacillus spp.
●	Bacillus subtilis
●	Others (give species names)
●	No chassis organism (please comment)

What risks could your chassis pose to you or your colleagues in the laboratory, or to your community or the environment if they escape the lab?
The general chassis organisms (*E. coli* and *S. cerevisiae*) are usually of no harm to human health and are well studied. This allows these organisms to be highly suitable as chassis organisms.

What experiments will you do with your organisms and parts?
We want to engineer a bacteriophage which is equipped with a chitin binding protein (safety form), that is able to travel via an insect vector. These chitin binding proteins will be tested on their binding affinity to select for the best option(s) possible.

This phage will be produced by the carrier bacterium (hypothetically a non-virulent Xanthomonas species, but we are doing this now in model organisms) and the phage will also carry the immune response triggering peptides (called MAMPs) on its chromosome.

These peptides will be tested in *Brassica oleracea* (in the lab), to see what/how the immune response (is) triggered.

We're going to regulate phage translation upon the presence of *X. fastidiosa* by hijacking the quorum sensing system of *X. campestris*. We will engineer a system with 2 different chimeric proteins able to detect the specific QS molecule and upon that, it will be able to activate translation of an Anti-CRISPR, which can relieve the repression of the phage genome by a dCas9.

What risks could arise from these experiments?
When working in the lab, there is basically always a chance of exposure to certain causes not mentioned by the protocol. For instance, vortexing Eppendorf tubes will most likely produce aerosols. Therefore, the provided Safety Data Sheets must be read when working with a chemical you're not familiar with. And especially, in this case, inhalation of this compound. When making solutions of chemicals it is necessary to work with extraction hoods or in a fume hood.

Workin sterile is preferably done in a laminar flow cabinet, but also working with a bunsen burner is possible. When working with a bunsen burner, no (latex) gloves should be worn to decrease the chance of severe burnings.

Plant inoculations will be done with needles. Therefore, you should take into account that working with needles takes along some risks like stinging yourself. It is not only a risk to yourself but also your colleagues and/or cleaners when not properly dealt with discarding these.

Furthermore, our experiments scheduled should limit the chance of bringing the environment to the lab or vice versa. We will only try to perform plant infection assays with the WT strain of *Xanthomonas* and the immune triggering response peptides will be injected after this infection manually, so no genetically engineered strains will be involved.

And, as a conclusion, we are aware that using microorganisms as a chassis or in general could potentially cause risks, even when this strain is classified as a Risk Group 1 organism. Each individual experiment should be prepared and assessed by a certain analysis to get an overview of all the potential risks available, biotic and abiotic.

Imagine that your project was fully developed into a real product that real people could use. How would people use it?

●	Our project is foundational / we do not have a specific real-world application in mind (Examples: library of standardized promoters, system for communication between cells)
●	Only in the lab (Examples: reporter strain for measuring the strength of promoters)
●	In a factory (Examples: cells that make a flavor chemical for food, cells that make biofuel)

●	In a consumer product that ordinary people buy (Examples: cells that clean your clothes, bread made with engineered yeast)
●	In agriculture / on a farm (Examples: cells that guard against pests, engineered rice plants, cells that promote growth of crop plants)
●	In a small enclosed device (Examples: a bio-sensing strip with cells that detect arsenic)
●	In the natural environment (Examples: cells that remove pollution from lakes, engineered forest trees that can resist drought)
●	To be used in the human body, or in food (Examples: anti-cancer bacteria, bread made with engineered yeast, engineered rice plants)
●	Other (Examples: bacteria that live on Mars, or a software project)

What safety, security or ethical risks would be involved with such a use?

In the field of synthetic biology/biotechnology, there are risks which need to be assessed in the case your project might be interesting as a real-life application. Our project might face the troubles of changes in host-specificity for the phages. This can be countered by keeping track of the latest most specific phages for *Xylella fastidiosa*. The genetically engineered phages might also recombine with the host bacterium. We hope to limit the effect of this by preferably using a protein native to *Xylella fastidiosa*.

Furthermore, we want to reduce or eliminate unnecessary phage production and immune activation of plants which can lead to plant death. Therefore, we're going to hijack a system present in a non-pathogenic *Xanthomonas*, making it able to sense *Xylella*'s presence and upon that, activate phage production. We also want to engineer a kill-switch that will kill the phage-carrying bacterium over-time.

But our main concern is that we are developing a phage which is able to uncontrollably spread itself. We counter this by the fact that bacteriophages are very specific. So, therefore, it is only infecting the plant pathogen *X. fastidiosa*. Although we do understand that spreading a GMO phage will raise some ethical questions. But then one of the main questions will be, is the cure worse than the disease? This is in our opinion a very interesting topic and we will elaborate more on this on the ethics pages of our wiki. For all these issues we have already spoken to experts to be able to gauge the risks associated with our project.

How will experts overseeing your project help to manage any of the risks you identified in this form?
Since we are working with a plant pathogen, other researchers present at the University were asked for their opinions and possible collaborations/training for our project, especially while working with *X. fastidiosa* and (infecting) plants.

Also for working with 'exotic' organisms, we arranged several meetings with the biosafety officer of our lab to see what is possible and if measures needed to be taken when working with these organisms.

Technicians are also very important, they need to know what we are working on, to oversee what risks might be involved for other experiments executed in the lab. And, of course, our supervisors will be there for the training in the specific lab. The technicians and supervisors are not only there for training, but they will also supervise and guide us in and out of the lab.

Furthermore, our whole team has taken a 2-hour introduction tour organized by the safety officer of the labs, covering all the ins and outs of safety regulations on these labs and the more general safety rules.

Please select the topics that you learned about (or will learn about) in your safety training

●	Lab access and rules (including appropriate clothing, eating and drinking, etc.
●	Responsible individuals (such as lab or departmental specialist or institutional biosafety officer)
●	Differences between biosafety levels
●	Biosafety equipment (such as biosafety cabinets)
●	Good microbial technique (such as lab practices)
●	Disinfection and sterilization
●	Emergency procedures
●	Transport rules
●	Physical biosecurity
●	Personnel biosecurity
●	Dual-use and experiments of concern

●	Data biosecurity
●	Chemicals, fire and electrical safety
●	We will not have safety training

Which work areas do you use / are you using to handle biological materials?

●	No lab work (e.g. software project)
●	Open bench
●	Biosafety cabinet (please note there are important differences between biosafety cabinets and laminar flow hoods / clean benches. iGEM encourages the use of biosafety cabinets but discourages the use of laminar flow hoods or clean benches. This Factsheet from the University of Massachusetts Amherst helps explain the differences.)
●	Specialist greenhouse
●	Specialist animal house
●	Specialist insect facility
●	Other work area. Please describe:
●	Unknown. Please comment:

What is the biosafety Level of your work space?

●	Not applicable as we have no lab component
●	Level 1 (low risk)
●	Level 2 (moderate risk)
●	Level 3 (high risk)
●	Level 4 (extreme risk)
●	Other biosafety level. Please describe:
●	We have several different lab spaces with different biosafety Levels. Please describe:<br>● We are working in level 1 and level 2 workspaces.

What other risk management tools will cover your work?

●	Accident reporting (measures to record any accidents)
●	Personal Protective Equipment (including lab coats, gloves, eye protection, etc)
●	An inventory control system (measures to track who has what materials and where they are)
●	Access controls (measures to control who can access your work spaces, or where materials are kept)
●	Medical surveillance (measures to find out if you get sick because of something you were using)
●	Waste management system (measures to make sure waste is not hazardous before it leaves your institution)
●	Special procedures or protocols that address safety or security
●	Others Please describe:

How will the rules, training, containment and other procedures and practices help to manage any of the risks you identified?

We do understand that our project has different parts which might raise questions among people. Therefore, we went out in the early stages of our project for help on our project. The talks with these experts have led to a better assessment of the risks we're encountering in our project. This might also result in collaborations between labs. Hereby, we are trying to make sure that the work we will do outside of our discipline, is carried out in the right way by the right people with the appropriate training.

Waste in our labs is discarded in a systemic way. Tripods carrying plastic bags are always discarded in the so-called 'autoclave bins'. These bins will be autoclaved when full, this can be done every day when necessary. Other waste bins are clear for the disposal of 'normal' waste.

2.2 Project #2: “Non-canonical AAs”

Describe the goal of your project: what is your engineered organism supposed to do? Please include specific technical details and names of important parts.

We aim at establishing a toolkit for the translational incorporation of non-canonical amino acids in *E. coli*. As an example for the successful incorporation of non-canonical amino acids we will develop a rapid test for prions and a new chromatography method for a mild elution of proteins. Moreover, we want to develop an *E. coli* strain which can retain unnatural base-pairs between isoG and isoC on expression vectors.

What organisms are you using as chasses in your project?

●	Escherichia coli (give names of all strains you are using)<br>● BL21, KRX, DH5alpha
●	Yeast (Saccharomyces cerevisiae)
●	Lactobacillus spp.
●	Bacillus subtilis
●	Others (give species names)<br>● The base isoguanine (isoG)
●	No chassis organism (please comment)

Do you plan to experiment with any other organisms, besides your chassis?

- We extracted DNA from *Pseudomonas putida* for a side project which aims at improving the spectrum of available selection markers for the community.

- We extract RNA from different tissues of *Croton tiglium L* for RNA-Seq. Our aim is the identification of the biosynthesis pathway leading to Isoguanosine.

Imagine that your project was fully developed into a real product that real people could use. How would people use it?

●	Our project is foundational / we do not have a specific real-world application in mind (Examples: library of standardized promoters, system for communication between cells)
●	Only in the lab (Examples: reporter strain for measuring the strength of promoters)
●	In a factory (Examples: cells that make a flavor chemical for food, cells that make biofuel)

●	In a consumer product that ordinary people buy (Examples: cells that clean your clothes, bread made with engineered yeast)
●	In agriculture / on a farm (Examples: cells that guard against pests, engineered rice plants, cells that promote growth of crop plants)
●	In a small enclosed device (Examples: a bio-sensing strip with cells that detect arsenic)
●	In the natural environment (Examples: cells that remove pollution from lakes, engineered forest trees that can resist drought)
●	To be used in the human body, or in food (Examples: anti-cancer bacteria, bread made with engineered yeast, engineered rice plants)
●	Other (Examples: bacteria that live on Mars, or a software project)<br>- An extended genetic code enables a huge variety of different products. We aim at demonstrating some of them during this summer.

What safety, security or ethical risks would be involved with such a use?

- *E.coli* is toxic if injected in the blood stream
- genes could be transferred to other strains
- if misused with malicious intent, antibiotic resistances could be combined in a pathogen

Please select the topics that you learned about (or will learn about) in your safety training

●	Lab access and rules (including appropriate clothing, eating and drinking, etc.
●	Responsible individuals (such as lab or departmental specialist or institutional biosafety officer)
●	Differences between biosafety levels
●	Biosafety equipment (such as biosafety cabinets)
●	Good microbial technique (such as lab practices)
●	Disinfection and sterilization
●	Emergency procedures
●	Transport rules

●	Chemicals, fire and electrical safety
●	We will not have safety training

Which work areas do you use / are you using to handle biological materials?

●	No lab work (e.g. software project)
●	Open bench
●	Biosafety cabinet
●	Specialist greenhouse
●	Specialist animal house
●	Specialist insect facility
●	Other work area. Please describe:<br>- Open bench and laminar flow cabinet
●	Unknown. Please comment:

What is the biosafety Level of your work space?

●	Not applicable as we have no lab component
●	Level 1 (low risk)
●	Level 2 (moderate risk)
●	Level 3 (high risk)
●	Level 4 (extreme risk)
●	Other biosafety level. Please describe:
●	We have several different lab spaces with different biosafety Levels. Please describe

Are there parts of your project which you think may have ethical, safety or security concerns that are not fully covered by current rules and standards?

- It is not clear how artificial DNA will be classified
- We need to do dual use considerations for antibiotic resistances

Who have you worked with to resolve any uncertainties or gaps in how you ensure the safety of your project and how difficult have they been to contact?

We regularly consulted our supervisors, who are very experienced and always very supportive. Furthermore, we asked our PIs about critical political or legal aspects. It was very easy to contact them.

2.3 Project #3: “Immune Escape”

Describe the goal of your project: what is your engineered organism supposed to do? Please include specific technical details and names of important parts.
Our project is going to engineer *Metarhizium anisopliae* (a kind of fungi that can kill cockroaches) by enhancing the ability of attachment and infection, helping them to evade immune cell recognition. And finally, suicide when they expose to outside the cockroaches.

Which whole organisms, including viruses and cell lines, are you planning to use or using in your project?

- *Metarhizium anisopliae* CQMa128, formerly known as *Entomophthora anisopliae* (basionym), is a fungus that grows naturally in soils throughout the world and causes disease in various insects.
- Cockroach, used to test the ability of wild-type fungus. And we will use the cuticle of the cockroach to detect the infection ability of our GMO.

What risks could these organisms pose to you or your colleagues in the laboratory, or to your community or the environment if they escape the lab?
It won't do harm to the environment. *Metarhizium anisopliae* exists in the wild in the environment and does not appear to infect humans or other vertebrates. The cockroaches we chose are from a regulated channel, which won't carry some specific germs.

What organisms are you using as chasses in your project?

●	Escherichia coli (give names of all strains you are using)
●	Yeast (Saccharomyces cerevisiae)
●	Lactobacillus spp.
●	Bacillus subtilis
●	Others (give species names)<br>● Metarhizium anisopliae CQMa128
●	No chassis organism (please comment)

M. anisopliae and its related species are used as biological insecticides to control a number of pests such as termites, thrips, etc. and its use in the control of malaria-transmitting mosquitoes is under investigation. *M.anisopliae* does not appear to infect humans or other vertebrates and is considered safe as an insecticide and as an acaricide. The microscopic spores are typically sprayed on affected areas.

What risks could your chassis pose to you or your colleagues in the laboratory, or to your community or the environment if they escape the lab
M. anisopliae does not appear to infect humans or other vertebrates and is considered safe as an insecticide. And we will ensure our organism will never expose outside the lab in the experimental stage. by using the suicide switch, our fungus will be safer.

What experiments will you do with your organisms and parts?
We propose to enhance the ability of our fungus.

We will extract the HsbA gene segment we want from *Beauveria bassiana* and link it to the constructed plasmid which is transferred into our strains, for which is able to promote the forming of adhesive spores.

In order to decompose the chitin on the cockroach's body surface, we will use the Bbchit1 gene segment from *Beauveria bassiana* and express it in our strains. By excreting chitinase, our strains can easily decompose and then drill into the cockroach's body.

It's necessary to avoid identification by the host's immune system. To do that, we will use an overexpression promoter to start over the MCL1 gene from our strains itself. That DNA helps our strains construct a molecular imitation in the host's body so that it can survive and cause illness.

With these three parts, our engineered strain is supposed to infect the cockroach and ultimately kill it.

To prevent genetically enhanced *M. anisopliae* from releasing to environment and killing other insects,we design a suicide switch with the ability to self-terminate after kill cockroaches. It contains promoter, tryptophan attenuator, MacF and fungal terminator. We will transfer this part into the fungus. And we will use the cuticle of the cockroach to detect the infection ability of our fungus.

What risks could arise from these experiments?
1. Our cockroaches may flee, which may cause some unexpected pollution.
2. The cockroaches may carry some causative germs, which may infect us in the experiment.

Imagine that your project was fully developed into a real product that real people could use. How would people use it?

●	Our project is foundational / we do not have a specific real-world application in mind (Examples: library of standardized promoters, system for communication between cells)

●	Only in the lab (Examples: reporter strain for measuring the strength of promoters)
●	In a factory (Examples: cells that make a flavor chemical for food, cells that make biofuel)
●	In a consumer product that ordinary people buy (Examples: cells that clean your clothes, bread made with engineered yeast)
●	In agriculture / on a farm (Examples: cells that guard against pests, engineered rice plants, cells that promote growth of crop plants)
●	In a small enclosed device (Examples: a bio-sensing strip with cells that detect arsenic)
●	In the natural environment (Examples: cells that remove pollution from lakes, engineered forest trees that can resist drought)
●	To be used in the human body, or in food (Examples: anti-cancer bacteria, bread made with engineered yeast, engineered rice plants)
●	Other (Examples: bacteria that live on Mars, or a software project)<br>● We mix the oil, spores, etc in a specific proportion and make the emulsifiable power.

What safety, security or ethical risks would be involved with such a use?
People may not easily accept the genetically modified product.

How will experts overseeing your project help to manage any of the risks you identified in this form?
All of us have received basic safety training from our course from our university. However, our instructors also used one week to make sure we got enough theoretical knowledge. And our team leaders, who participated iGEM last year, also have led us to do a series of experiments to make sure we get enough practical experiences.There are three main guidelines:
1. Hardware: There should be essential experiment equipment, which has complete function.
2. People: All the people who operate experiments should be trained strictly.
3. Software: Complete regulation systems should be set up.

The three guidelines supplement each other. These guidelines help us achieve two basic but very important goals.

1. Make sure all people doing the experiments will not be infected.
2. Make sure the environment will not be polluted.

Please select the topics that you learned about (or will learn about) in your safety training:

●	Lab access and rules (including appropriate clothing, eating and drinking, etc.
●	Responsible individuals (such as lab or departmental specialist or institutional biosafety officer)
●	Differences between biosafety levels
●	Biosafety equipment (such as biosafety cabinets)
●	Good microbial technique (such as lab practices)
●	Disinfection and sterilization
●	Emergency procedures
●	Transport rules
●	Physical biosecurity
●	Personnel biosecurity
●	Dual-use and experiments of concern
●	Data biosecurity
●	Chemicals, fire and electrical safety
●	We will not have safety training

Which work areas do you use / are you using to handle biological materials?

●	No lab work (e.g. software project)
●	Open bench
●	Biosafety cabinet (please note there are important differences between biosafety cabinets and laminar flow hoods / clean benches. iGEM encourages the use of biosafety cabinets but discourages the use of laminar flow hoods or clean benches. This Factsheet from the University of Massachusetts Amherst helps explain the differences.)
●	Specialist greenhouse

●	Specialist animal house
●	Specialist insect facility
●	Other work area. Please describe:
●	Unknown. Please comment:

What is the biosafety level of your work space?

●	Not applicable as we have no lab component
●	Level 1 (low risk)
●	Level 2 (moderate risk)
●	Level 3 (high risk)
●	Level 4 (extreme risk)
●	Other biosafety level. Please describe:
●	We have several different lab spaces with different biosafety Levels. Please describe

How will the rules, training, containment and other procedures and practices help to manage any of the risks you identified?

The fungus we used exists in soil in the wild, and does not infest humans or other vertebrates. We are well trained in experiments, which help us get rid of the infection from cockroaches. We will centralize disposal of the waste and control the pollution. All of the fungus and other products will be sterilized by autoclave after the experiment.

2.4 Project #4: “Psilocybin”

Describe the goal of your project: what is your engineered organism supposed to do? Please include specific technical details and names of important parts.

We are attempting to move the biosynthetic pathway for psilocybin from the mushroom *Psilocybe cubensis* into *Escherichia coli*. We will be cloning four metabolic enzymes required for the conversion of tryptophan to tryptamine to 4-hydroxytryptamine to nor-baeocystin to baeocystin to psilocybin. Psilocybin is a psychedelic drug that has shown efficacy in the treatment of affective and anxiety disorders such as depression and post-traumatic stress disorder. A reliable, scalable, and efficient method of psilocybin production is necessary to enable further clinical research and provide a cost-effective source of the pharmaceutical, if it becomes more widely used as a medicine.

Which whole organisms, including viruses and cell lines, are you planning to use or using in your project?

Escherichia coli TOP10

- Genotype: F- mcrA delta(mrr-hsdRMS-mcrBC) phi80lacZdeltaM15 deltalacX74 nupG recA1 araD139 delta(ara-leu)7697 galE15 galK16 rpsL(StrR) endA1 lambda-

What risks could these organisms pose to you or your colleagues in the laboratory, or to your community or the environment if they escape the lab?

E. coli containing the psilocybin biosynthetic pathway poses additional risks, as follows.

1. Psilocybin is a powerful psychedelic (hallucinogenic) drug and thus must be handled and used with care (ie. under appropriate medical supervision). Ingestion of a sufficient number of recombinant *E. coli* cells containing this pathway and metabolite would therefore lead to symptoms similar to those of ingesting 'magic' mushrooms.

2. There are legal risks involved if our recombinant *E. coli* cells were distributed without appropriate permissions or otherwise misused, since psilocybin is illegal to possess in most countries.

3. The final recombinant *E. coli* cells from our project will contain two plasmids, each with different resistance genes (kanamycin-R and ampicillin-R). If released to the environment, these plasmids could contribute to the growing problem of antibiotic resistance.

What organisms are you using as chasses in your project?

●	Escherichia coli (give names of all strains you are using)<br>- TOP10
●	Yeast (Saccharomyces cerevisiae)
●	Lactobacillus spp.
●	Bacillus subtilis
●	Others (give species names)
●	No chassis organism (please comment)

What risks could your chassis pose to you or your colleagues in the laboratory, or to your community or the environment if they escape the lab?

Escherichia coli TOP10 is a K-12 derived strain. K-12 been assessed by the United States Environmental Protection Agency (EPA) as having a "very low likelihood of acting as a pathogen of humans or animals". The further mutations in K-12 that led to the TOP10 strain further weaken the organism and make it even less likely to survive in the environment or in the human intestine.

What experiments will you do with your organisms and parts?

We will test the function of each of the four psi genes individually in *E. coli* by cloning into pET28b, overexpression and purification of the enzymes as 6xHis tag fusion proteins, and testing biochemical activity on the corresponding substrates (tryptophan, tryptamine, 4-hydroxytryptamine, nor-baeocystin, baeocystin), with analysis via LC-MS.

Then we will clone the genes together for final expression in *E. coli*. This will be done by adding psiD, psiK and psiM into the vector pUS250, and the psiH gene into the plasmid pCWori-CPR, which already contains the required partner enzyme for psiH (human cytochrome p450 reductase). These two plasmids have different replication origins and resistances, and are compatible in E.coli. The final recombinant strain will be tested for psilocybin production from tryptophan by LC-MS.

Based on previous literature on cytochrome p450 enzymes, we expect functional expression of PsiH in *E.coli* to be difficult. Therefore we will test methods for codon harmonisation using different versions of a fluorescent test protein (VVD-36 from Neurospora crassa). The results of these tests will inform codon harmonisation of the psiH gene, and hopefully improve its function in *E.coli*.

What risks could arise from these experiments?

AGAROSE GEL ELECTROPHORESIS

- Electric shock from electrophoresis apparatus
- Burns from hot agarose
- Explosion in microwave if a sealed bottle is heated
- Cancer/burns risk from UV light

USE OF BIOSAFETY II CABINETS

- Possibility of release of GMOs to the environment due to incorrect use of cabinet
- Possibility of fire in the biosafety cabinet due to inappropriate use
- Possibility of burns to eyes or skin from UV light in cabinet
- Fire hazard due to use of ethanol to sterilise the cabinet

USE OF BUNSEN BURNERS

- Bunsen flame can cause burns
- Bunsen flame can start fires if flammable materials are nearby

CENTRIFUGATION

- Rotor imbalance can cause catastrophic failure of centrifuge (explosion), injuring user
- Rotor can fail due to excessive speed, causing catastrophic failure of centrifuge, injuring user
- Rotor can fail due to extensive wear and tear, causing catastrophic failure of centrifuge, injuring user
- Centrifuge tubes can break in rotor due to excessive wear and tear or inappropriate use, causing imbalance of rotor, catastrophic failure of centrifuge, injuring user

WORKING WITH CORROSIVES

- Contact/Absorption: corrosive liquids have high potential to cause serious burns and irritation to skin and eyes
- Inhalation: corrosive gases/vapour can damage lining of the lungs
- Ingestion: severe irritation (nausea, vomiting and diarrhoea) and permanent damage to gastrointestinal tract
- Other hazards: may react with other substances and generate/evolve heat

DNA EXTRACTION

- Eye damage if corrosive chaotropic salts contact eyes
- Poisoning if chaotropic salts ingested

- Skin damage if chaotropic salts contact bare skin
- Environmental damage if chaotropic salts disposed of incorrectly
- Environmental damage if recombinant DNA is disposed of incorrectly.
- Injury due to incorrect use of centrifuge
- Fire or electrocution due to centrifuge malfunction

USE OF FUME HOOD

- Harmful vapours (toxic, corrosive, stench) may escape from hood if it is not set up correctly
- Large objects can disrupt the airflow within the fumehood
- Use of a Bunsen burner or other ignition source in hood can lead to a fire

USE OF MICROWAVE

- Over heated liquids resulting in burns
- Explosion from heated closed vessels

WORKING WITH RISK GROUP 1 MICROORGANISMS

- Hazards are infection of lab worker, infection of other people, infection of plants or animals, and escape of the microorganism into the environment. Note that these hazards are very minimal for Risk Group 1 microbes.

SDS-PAGE (POLYACRYLAMIDE GEL ELECTROPHORESIS)

- Acrylamide used to make gel is a potent neurotoxin. Potential for inhalation, skin/eye contact or ingestion with harmful effects.
- SDS (sodium dodecyl sulphate) is harmful by inhalation, ingestion, skin or eye contact.
- TEMED used for making gel. Potential for inhalation, ingestion, skin or eye contact with harmful effects. TEMED produces a strong stench and is a corrosive alkali. TEMED is also flammable.
- APS used for making gel. Potential for inhalation, ingestion, skin or eye contact with harmful effects. APS is a strong oxidizer and is corrosive.
- Mercaptoethanol in the buffer used for denaturing protein samples. Potential for inhalation, ingestion, skin or eye contact with harmful effects. Mercaptoethanol produces a strong stench, and is toxic.
- Electrophoresis. Potential for serious electrical shock or electrocution due to leaking chamber, faulty or corroded electrode cables, or faulty power supply.

SYNTHESIS OF RESTRICTED AND PSYCHOACTIVE SUBSTANCES

- Accidental exposure and dosage with psilocybin
- Legal problems (arrest, jail etc)

Imagine that your project was fully developed into a real product that real people could use. How would people use it?

●	Our project is foundational / we do not have a specific real-world application in mind (Examples: library of standardized promoters, system for communication between cells)
●	Only in the lab (Examples: reporter strain for measuring the strength of promoters)
●	In a factory (Examples: cells that make a flavor chemical for food, cells that make biofuel)
●	In a consumer product that ordinary people buy (Examples: cells that clean your clothes, bread made with engineered yeast)
●	In agriculture / on a farm (Examples: cells that guard against pests, engineered rice plants, cells that promote growth of crop plants)
●	In a small enclosed device (Examples: a bio-sensing strip with cells that detect arsenic)
●	In the natural environment (Examples: cells that remove pollution from lakes, engineered forest trees that can resist drought)
●	To be used in the human body, or in food (Examples: anti-cancer bacteria, bread made with engineered yeast, engineered rice plants)
●	Other (Examples: bacteria that live on Mars, or a software project)

What safety, security or ethical risks would be involved with such a use?

Psilocybin is a restricted or illegal substance in most countries, and has security and safety risks such as theft or illicit use. Usage of the compound outside of a controlled medical environment (ie. "recreational" use) is hazardous due to the strongly mind-altering nature of this drug - e.g. driving a car under the influence of psilocybin would be unwise (and illegal).

There are also possible risks with possession or supply of recombinant cells that contain the psilocybin biosynthetic pathway, or plasmids that encode the biosynthetic pathway - these appear to be legal 'grey areas' and will be the subject of some of our human practices work.

How will experts overseeing your project help to manage any of the risks you identified in this form?
Oversight of GMO work at our institution is done by the Institutional Biosafety Committee (IBC). The proposed work has been approved by the IBC as an exempt (low risk) dealing within the meaning of the Gene Technology Regulations 2001, which is regulated at the national level by the Office of the Gene Technology Regulator (OGTR).

Authority to manufacture, produce, and possess the prohibited drug psilocybin, and some potentially psychoactive intermediate metabolites (psilocin, baeocystin, norbaeocystin) has been provided to our institution's lab by the regionalMinistry of Health, by authority of the Deputy Chief Pharmacist, pursuant to the Drug Misuse and Trafficking Act 1985. The Dean of Science and the head of the university's Work Health and Safety Unit are aware of the project and have given their approval for it to proceed.

The project is proceeding under the close supervision of our supervisor, an Associate Professor of Microbiology, who has extensive familiarity with the procedures and practices to be used in the project, and also has 10 years experience as a departmental safety officer and chair of the school safety committee.

Please select the topics that you learned about (or will learn about) in your safety training

●	Lab access and rules (including appropriate clothing, eating and drinking, etc.
●	Responsible individuals (such as lab or departmental specialist or institutional biosafety officer)
●	Differences between biosafety levels
●	Biosafety equipment (such as biosafety cabinets)
●	Good microbial technique (such as lab practices)
●	Disinfection and sterilization
●	Emergency procedures
●	Transport rules
●	Physical biosecurity
●	Personnel biosecurity

●	Dual-use and experiments of concern
●	Data biosecurity
●	Chemicals, fire and electrical safety
●	We will not have safety training

Which work areas do you use / are you using to handle biological materials?

●	No lab work (e.g. software project)
●	Open bench
●	Biosafety cabinet (please note there are important differences between biosafety cabinets and laminar flow hoods / clean benches. iGEM encourages the use of biosafety cabinets but discourages the use of laminar flow hoods or clean benches. This Factsheet from the University of Massachusetts Amherst helps explain the differences.)
●	Specialist greenhouse
●	Specialist animal house
●	Specialist insect facility
●	Other work area. Please describe:
●	Unknown. Please comment:

What is the biosafety Level of your work space?

●	Not applicable as we have no lab component
●	Level 1 (low risk)
●	Level 2 (moderate risk)
●	Level 3 (high risk)
●	Level 4 (extreme risk)
●	Other biosafety level. Please describe:
●	We have several different lab spaces with different biosafety Levels. Please describe:

What other risk management tools will cover your work?

●	Accident reporting (measures to record any accidents)
●	Personal Protective Equipment (including lab coats, gloves, eye protection, etc)
●	An inventory control system (measures to track who has what materials and where they are)
●	Access controls (measures to control who can access your work spaces, or where materials are kept)
●	Medical surveillance (measures to find out if you get sick because of something you were using)
●	Waste management system (measures to make sure waste is not hazardous before it leaves your institution)
●	Special procedures or protocols that address safety or security
●	Others Please describe:

How will the rules, training, containment and other procedures and practices help to manage any of the risks you identified?

We have received training in safe work practices, and our entire project group has completed an in-person building and laboratory induction with a laboratory manager, as well as an online induction that covers use of equipment, hazard reduction and identification, and evacuation and safety procedures. Our group has read and signed all standard operating procedures that are relevant to work for this project.

As we are working in a PC2 space, our work necessitates use of PPE (lab gown, enclosed impermeable footwear, safety glasses, gloves) at all times. In addition, our lab incorporates standardised decontamination and waste management procedures. Chemical and biological waste is identified, treated and inactivated as appropriate. Risk of environmental release is low, as all biologicals are subject to decontamination, and chemical waste is decanted into containers with contents labelled, dated and disposed of by the laboratory Research Support Team.

3 Supplemental methods: recruitment, participation, data cleaning, and statistics

We surveyed 122 recent iGEM participants and 34 biorisk experts between July and November 2021. iGEM participants were recruited using the iGEM website, newsletter, and Slack group. Experts were drawn mostly from an email advertisement to the iGEM Safety and Security committee email list, who regularly review iGEM projects for biosafety and biosecurity risks, though we also extended invitations to experts who have worked on specific DURC risk assessment projects (the Dual Use Quickscan,[1] Malice Analysis,[2] and attendees of a US Government stakeholder engagement workshop on the DURC policy.[3])

Each participant was first invited to sign a consent form and fill out a short background survey. Participants were rejected from participating if they said that they were under 18 years old. At the end of the background survey, they were provided with links to DURC risk assessment forms for all four of the iGEM projects to be reviewed, and they were invited to complete as many as they wanted. To ensure that participants were equally likely to evaluate any of the four projects, we mapped each project to a set of three months out of the calendar year and then told participants to start with the project that corresponded to their birth month (starting with Project #1 if they were born in January, February, or March, and so on). Each project review typically took between 20 minutes and 1 hour.

After collecting all data, we cleaned and filtered the responses as follows. Across the combined expert and student background survey data, we removed the following rows of data:

- 34 responses without an email address, because we would be unable to link them to the evaluation form responses. These were all entirely blank responses created by someone opening and immediately closing the survey.
- 2 test responses created by the authors.
- 2 incomplete responses from participants that failed to say that they were over 18 and were rejected from the remainder of the survey.

Across the combined expert and student DURC risk assessment form data (with each row corresponding to a single project evaluation), we removed the following rows of data:

- 6 test responses created by the authors.

---

[1] Vennis, I., Schaap, M., Hogervorst, P., et al. (2021). *Dual-use quickscan: A web-based tool to assess the dual-use potential of life science research*. Frontiers in Bioengineering and Biotechnology 9.573 doi:10.3389/fbioe.2021.797076

[2] Engineering Biology Research Consortium. *Malice Analysis: Assessing Biotechnology Research for Security Concerns.* [cited 2024 Jun 06] https://ebrc.org/malice-analysis/

[3] Evans, S., Greene, D., Hoffmann, C., and Lunte, S. (2021). *Stakeholder Engagement Workshop on the Implementation of the United States Government Policy for Institutional Oversight of Life Sciences Dual Use Research of Concern: workshop report*. Tech. rep. doi:10.2139/ssrn.3955051

- 31 responses from participants who signed up to participate as students, but had not been a student or student leader on an iGEM team since 2018. These included iGEM team faculty leaders and other instructors. We verified this by cross-referencing the email addresses provided by participants with the official iGEM user database.
- 37 mostly-incomplete responses that were duplicate reviews of the same project by the person identified by a previously-observed email address. We retained the first email-project combination and removed any later duplicates.

Our statistical approach was as follows.

- For analyses that compare expert and student ratings of projects, we used mixed-effects models when possible to capture variance between reviewers and between projects.[4] In each case, we report the estimated effect of expertise with chi-square test results from an ANOVA that compares a null model, with random effects of reviewer and project, to an augmented model that has an additional fixed effect of expertise.[5]
- However, in some analyses, mixed effect models did not converge. This is likely because some reviewers only evaluated one project, and thus any estimate of the effect of the reviewer would be conflated with an estimate of the effect of the project that they reviewed. In these situations, we followed pre-existing practice and averaged the value of the outcome variable across all four projects for each participant and then reported the results of a simple linear model comparing experts and novices on their average scores. This approach is less statistically powerful than a mixed-effects model because it fails to model variance between projects, but it preserves the assumption of independence between data points because each point represents a single participant.[5]
- To judge whether the variances of two distributions are significantly different from one another, such as expert and novice ratings of project risks, we applied a permutation test.[6] We relabeled each data point with random "Expert" and "Student" labels 10,000 times and calculated the observed difference between expert and student variances each time, then calculated the percentage of our simulations that were greater than the empirically-observed value due to chance.
- Finally, all t-tests are two-sided, and all tests of individual projects have a Bonferroni correction of n = 4 applied (for four projects) with a threshold of statistical significance threshold of .05/4 = 0.0125.

---

[4] Gelman, Andrew, and Jennifer Hill. Data analysis using regression and multilevel/hierarchical models. Cambridge university press, 2007.
[5] Winter B. *Linear models and linear mixed effects models in R with linguistic applications*. arXiv preprint. 2013. http://arxiv.org/pdf/1308.5499.pdf
[6] Welch WJ. *Construction of permutation tests*. Journal of the American Statistical Association. 1990; 85(411):693-698.

4 Background survey

4.1 Introduction

This survey should only take a minute. Please note that there are no right or wrong answers to any of the questions! We welcome your honest responses.

4.2 Consent form

Dear Participant:

You are invited to participate in a research study that explores how people involved with the iGEM competition evaluate the potential safety and security risks of iGEM projects. If you decide to participate, you will participate in one or more online surveys in which you may be asked to answer questions about your background, share your opinions about various topics related to life-science research risks, and/or read texts and write short responses. Some surveys may ask you to evaluate current or former iGEM projects for risks!

Each survey takes between 5 minutes and 90 minutes to complete, and you may be invited to complete several surveys. Depending on how many you would like to do, all of the surveys could take between 5 minutes and 10 hours at the absolute most, but very likely close to 4 hours. Please keep in mind that you can stop at any time and space them out! We think that these are more fun and interesting than typical surveys because you will be thinking deeply about tricky questions in biology and risk assessment.

Once we receive your survey responses, we may link them with publicly available information about your iGEM team, your project wiki, and your safety forms, if you are an iGEM participant. This will let us answer questions like "How do people who work on different kinds of projects rate project risks differently?" Once we do this, we will remove any identifying information to make your answers de-identified. After that, we may also securely share your de-identified survey responses with the iGEM Foundation so that they can learn how to do risk assessment more effectively.

You will not be paid for participation, but there are also no risks associated with participation. Participation is completely optional, and your decision about whether or not to participate in this study will not be counted against you in any way. However, the knowledge gained from this study can help us make iGEM better and help you learn about human practices, so we hope that you will be eager to participate.

Please understand that your participation is voluntary, and you have the right to withdraw your consent or stop participating at any time without any. You also have the right to refuse to answer particular questions. If you mention evidence of abuse or neglect during your responses, we are required by law to report any concerns for your safety. Your individual privacy will be maintained in all published data resulting from the study, unless you provide us with explicit consent to share your identity in publications.

If you have any questions, concerns or complaints about this research study, its procedures, risks and benefits, please contact the Protocol Director, Daniel Greene, at dkgreene@stanford.edu or by phone at 570-856-1216.

If you are not satisfied with how this study is being conducted, or if you have any concerns, complaints, or general questions about the research or your rights as a participant, please contact the Stanford Institutional Review Board (IRB) to speak to someone independent of the research team at (650)-723-2480 or toll free at 1-866-680-2906. You can also email irbnonmed@stanford.edu or write to the Stanford IRB, Stanford University, Stanford, CA 94305-5401.

By writing your name, email address, and the current date below, you acknowledge that you are at least 18 years old, you have read the text above, and you consent to participate in the study.

(name)
(email address)
(current date)
Are you at least 18 years old? (Yes/No)

4.3 Survey body

What is the highest educational degree that you have completed?

- High school degree or equivalent
- Bachelor's degree (BA, BS, etc.)
- Master's degree (MA, MS, etc.)
- Terminal degree (PhD, MD, JD, etc.)

How many years of undergraduate college have you completed?

- 0
- 1
- 2
- 3
- 4
- 5 or more

If you added up all the time you've spent doing the following kinds of synthetic biology work, roughly how much time would it be?

- Wet lab synthetic biology (e.g. cloning, fermentation, plant synbio)
  - Basically none
  - Days
  - Months
  - Years
- Dry lab synthetic biology (e.g. bioinformatics, math modelling)
  - (same options as above)
- Broader aspects of synthetic biology (e.g. human practices, sociology of science, risk analysis)
  - (same options as above)

Have you participated in any training on "dual-use assessment" or "DURC" issues from iGEM?

- Yes
- No
- Not sure

Before you participated in this project, how familiar were you with the concept of DURC (Dual-Use Research of Concern)?

- Not familiar at all
- Slightly familiar
- Moderately familiar
- Very familiar
- Extremely familiar

In your opinion, what percentage of synthetic biology research being done today has the potential to be DURC, using the definition given in the original email?
As a reminder, a project would be considered DURC if, based on current understanding, it can be reasonably anticipated to provide knowledge, information, products, or technologies that could be directly misapplied to pose a significant threat with broad potential consequences to public

health and safety, agricultural crops and other plants, animals, the environment, materiel, or national or global security.

- (Slider scale, 0-100%)

Do you think that synthetic biology researchers are generally too cautious about the potential risks of their work, or too careless about those risks, or somewhere in between?

- Way too cautious
- Moderately too cautious
- Slightly too cautious
- Neither too cautious nor too careless
- Slightly too careless
- Moderately too careless
- Way too careless

How willing are you to take risks, in general?

- (0-10 scale, 0 marked as “Not at all willing”, 10 marked as “Extremely willing”)

5 DURC risk assessment form

5.1 Explanatory note from the authors

We modified the questions from the US Government's 2014 DURC policy companion guide for academic institutions to fit a survey format, and occasionally to harmonize language across questions, but their order was roughly maintained. Because we anticipated differences in prior knowledge between participants, we tried to define terms and clarify exactly what we were expecting from each question. We recruited two iGEM alumni to pilot the survey and modified it for additional clarity based on their feedback.

Initial interviews suggested that expert participants were less willing to invest substantial time on reviews than were students, so we streamlined the DURC risk assessment survey for expert participants by attempting to remove questions that we thought were less essential and streamlining open-response questions into brief prompts.

The following is a summary of the differences between the student and expert versions of the DURC risk assessment form:

- The initial quantitative evaluations of DURC risk and expected benefits were excluded from the expert form.
- The Likert-scale question about the overall expected magnitude of harm was retained in the expert form, but the related open-response questions in that section (nature of harm, ability to manage harm, magnitude of harm) were either excluded or condensed into brief prompts to inform the Likert-scale question.
- Similarly, the Likert-scale question about the overall expected likelihood of harm was retained in the expert form, but the related open-response and Likert-scale questions in that section (skill needed to cause harm, difficulty in acquiring resources, immediacy of harm potential) were either excluded or condensed into brief prompts to inform the Likert-scale question.
- The open-response questions related to the magnitude and time frame of benefits were replaced with prompts for consideration to inform their corresponding Likert-scale questions.
- The questions about use of additional resources and time spent on additional research were excluded.

The student form is presented below in its entirety.

5.2 Introduction to assessment form

Thank you for volunteering to do a dual-use assessment of an iGEM project!

Please take your time and read everything carefully. This form will walk you through everything you need to know.

We expect the assessment to take roughly one hour.

What's a single email address where we can send a copy of your responses? [open response]

May we contact you at that email address if we have follow-up questions? [yes/no]

5.3 Get set up

Here is a link to a form describing the project that you will review. Please open this link in a new tab or window, and keep it open as you fill out this form. If you need to find it again, you can always come back to this page.

Next, please read the entire project form. This should take about 10-20 minutes. We also recommend opening a blank text document or keeping a piece of paper around if you want to take notes as you read. When you're done, come back here and click to continue.

5.4 Initial questions about the project

After reading the provided materials, how well do you think you understand this project?

- Not at all well
- Slightly well
- Moderately well
- Very well
- Extremely well

How familiar are you with the topic or problem area addressed by this project?

- Not at all familiar
- Slightly familiar
- Moderately familiar
- Very familiar

- Extremely familiar

How familiar are you with the synthetic biology and/or engineering methods used in this project?
- Not at all familiar
- Slightly familiar
- Moderately familiar
- Very familiar
- Extremely familiar

Overall, how excited are you about the knowledge, information, products, or technologies being developed by this project?
- Not at all excited
- Slightly excited
- Moderately excited
- Very excited
- Extremely excited

Overall, how much risk do you think that the knowledge, information, technology, or products from this project pose to public health and safety, agricultural crops and other plants, animals, the environment, materiel, or national or global security.?
- Very low risk
- Moderately low risk
- Moderate risk
- Moderately high risk
- Very high risk

Overall, how large or small are the expected benefits of this project?
- Very small
- Moderately small
- Moderate
- Moderately large
- Very large

5.5 Part 1 of 3 - Risk assessment

This next section comprises the majority of the assessment. You will be assessing the project for dual-use risks. As a reminder, here is the definition again:

What is a dual-use assessment?

We are asking you to assess how much a project involves dual-use research of concern, or DURC for short.

A project would be considered DURC if, based on current understanding, it can be reasonably anticipated to provide knowledge, information, products, or technologies that could be directly misapplied to pose a significant threat with broad potential consequences to public health and safety, agricultural crops and other plants, animals, the environment, materiel, or national or global security.

This definition is important, so let's break down each part:

- "reasonably anticipated":
  - This means that the risk is not extremely unlikely or speculative.
- "knowledge, information, products, or technologies":
  - This means that the project's physical materials or products could cause harm, or that its *ideas* could be used to cause harm.
- "directly misapplied":
  - This means that, without a lot of additional work, the project could cause harm, whether by accident (e.g. a lab escape of the project) or deliberately (e.g. a bad actor repurposing the project to do harm).
- "significant threat with broad potential consequences":
  - This means that the harm caused by the project could be at a large scale, either harming many many things or a few very important things, and ultimately affecting many people's lives.
- "public health and safety, agricultural crops and other plants, animals, the environment, materiel, or national or global security":
  - Public health and safety, agricultural crops and other plants, animals, and the environment are straightforward. "Materiel" here means the supplies and tools used for society or the economy to function, like construction materials and electrical equipment. "National or global security" means not just military defense, but also the social and political stability of a country or countries.

A DURC project could raise concern through its potential to accidentally cause significant harm (for example, through escape to the environment) or to be misused to cause significant harm (for example, if someone outside of iGEM studied the project wiki and adapted the ideas to make a weapon).

All life sciences research has some potential for harm, but not all of it is considered DURC. For example, a laboratory-acquired *E. coli* infection or an accidental cut from broken glass would not be considered harm on the scale of DURC.

We're not asking you to do a binary classification of whether a project is DURC or not. Instead, we want to know to what extent you think it involves these concerns.

Let's get started.

5.6 Potential harm from misuse

When considering the potential harm from the misuse of this project, think broadly about impacts to public health and safety, agricultural crops and other plants, animals, the environment, materiel, or national or global security.

5.6.1 Nature of potential harm

Consider the nature and scope of the potential harm that might result from misuse of the project. *All else equal, information that could be misused to harm numerous sectors of society or the environment may be of greater concern.*

What areas of society or the environment could potentially be harmed by misuse of the knowledge, information, technologies, or products from this project, and how? [open response]

5.6.2 Magnitude of potential harm

Consider the magnitude of the potential harm.
All else equal, research or research information that could be misused to cause severe harm may be of greater concern.

How severe do you think the potential harm could be to society or the environment? Why? [open response]

5.6.3 Ability to manage potential harm

Consider the available measures to manage potential harm.
Adequate risk management measures may help to decrease concern. These measures may include countermeasures like drugs, biological products, public health practices, pesticides; devices intended for diagnosis, detection, mitigation, prevention, or treatment; or containment and surveillance approaches.

Are there currently any measures to help manage the potential harm? Are they readily available? [open response]

5.6.4 Overall potential harm of misuse

Assuming that this project did cause harm, how much harm *could* the knowledge, information, technology, or products from the project cause to public health and safety, agricultural crops and other plants, animals, the environment, materiel, or national or global security?

- Not much harm at all
- A small amount of harm
- A moderate amount of harm
- A large amount of harm
- A massive amount of harm

Please explain how you chose your answer below: [open text response]

5.7 Likelihood of misuse

5.7.1 Technical skill and physical resources required to cause harm

Consider the technical skill and/or physical resources that would be needed to misuse this project.
The risk of misuse may be lower for knowledge, information, technologies, or products that would be expensive, difficult to get, or that require a high degree of technical skill to facilitate such misuse.

How much technical skill would it take to cause harm using the knowledge, information, technologies, or products from this project?

- No technical skill at all
- A little technical skill
- A moderate amount of technical skill
- A lot of technical skill
- A great deal of technical skill

Please explain your answer: [open response]

How expensive or difficult to acquire are the physical resources (e.g. materials and equipment) needed to cause harm using the knowledge, information, technologies, or products from this project?

- Not expensive or difficult at all
- A little expensive or difficult
- Moderately expensive or difficult
- Very expensive or difficult
- Extremely expensive or difficult

Please explain your answer: [open response]

5.7.2 Immediacy of misuse

Consider whether the project in question could be directly misused to harm society or the environment.
The risk of misuse may be higher for research information that can be directly misused than for research information that requires significant additional scientific advances to facilitate its misapplication.

Can the knowledge, products, information, or technologies generated from the project be directly misused, without further scientific advances?

- Yes
- No, but the project could be misused when combined with information that already exists
- No, but the project could perhaps be misused with significant further scientific advances
- No, there is no obvious way for the project to be misused

Please explain your answer: [open response]

5.7.3 Overall likelihood of misuse

Ignoring the scope of potential harm, how likely is it that the knowledge, information, technology, or products from the research would be misused to harm society or the environment?

- Not at all likely
- Slightly likely
- Moderately likely
- Very likely
- Extremely likely

Please explain how you chose your answer below: [open text response]

5.8 Overall risk

Overall, combining your judgments of the potential harms of misuse with the likelihood of misuse…

How much risk do you think that the knowledge, information, technology, or products from this project pose to public health and safety, agricultural crops and other plants, animals, the environment, materiel, or national or global security?

- Very low risk
- Moderately low risk
- Moderate risk
- Moderately high risk
- Very high risk

How confident are you about your assessment of the overall risk?

- Not at all confident
- Slightly confident
- Moderately confident
- Very confident
- Extremely confident

Please use this space for any additional optional comments on your risk assessment. [open response]

5.9 Part 2 - Benefit assessment

In this next section, you will consider the potential benefits of the project. After all, the "dual-use" part of "dual-use research of concern" refers to its potential for both risks and benefits.

5.9.1 Magnitude

Consider the nature and scope of the potential benefits (e.g. to human health, scientific fields, populations of plants or animals) that might result from use of the project.

What areas of society or the environment could potentially benefit from use of the knowledge, information, technologies, or products from this project, and how? [open response]

Assuming that this project did realize its potential benefits, how large or small is the magnitude of those benefits?

- Very small
- Moderately small
- Moderate
- Moderately large
- Very large

5.9.2 Likelihood

Consider the time frame in which benefits from the research might be realized. *Information that can be positively used in the near term may be considered to be of greater benefit.*

Looking forward from the team's completion of the project, how soon might the potential benefits be realized?

- Less than 1 year
- 2 to 5 years
- 6 to 10 years
- 11 to 50 years
- 51+ years
- Never

How likely is this project to create its potential benefits, whether it's carried on by the iGEM team or by others in the scientific community?

- Not at all likely
- Slightly likely
- Moderately likely
- Very likely
- Extremely likely

5.9.3 Overall

Overall, considering <u>both</u> the potential benefits and likelihood of use… How large or small are the <u>expected</u> benefits of this project?

- Very small
- Moderately small
- Moderate
- Moderately large
- Very large

How confident are you about your assessment of the overall benefit?
- Not at all confident
- Slightly confident
- Moderately confident
- Very confident
- Extremely confident

Please use this space for any additional optional comments on your benefits assessment. [open response]

5.10 Part 3 of 3 - Recommended risk management

Almost done! In this section, you will recommend strategies that the team should take to manage the dual-use concerns in their project.

Your job here is to strike a balance two different goals:

- The first goal is preventing harm, whether to the iGEM team, their community, or the environment. If an iGEM project caused serious harm—for example, by making someone sick or damaging the environment—it would threaten the future of both the iGEM competition and the field of synthetic biology.
- The second goal is making scientific progress. Steps to mitigate risks can create real barriers to scientific progress. If iGEM teams are asked to do excessively difficult things to manage risks, then they might have to give up their projects.

Different projects have different risks. You are free to recommend no changes to any project if you think that none are needed, or many changes if you think that they are all needed. Thank you for your help!

- Should the team complete additional training that is relevant to the specific risks of the project? This might include: special training for handling certain strains or chemicals, or seeking expert advice about the risks in the project.
    - This strategy is not necessary
    - This strategy doesn't seem necessary, but the team could consider it
    - The team should seriously consider this strategy

    - The team should not continue the project without this strategy

- Should the team monitor their work more closely and report more frequently to iGEM? This might include: providing ongoing regular reports to iGEM, or identifying milestones that require the project to be reviewed again by iGEM before proceeding.
    - This strategy is not necessary
    - This strategy doesn't seem necessary, but the team could consider it
    - The team should seriously consider this strategy
    - The team should not continue the project without this strategy

- Should the team implement additional biosafety measures? This might include: using additional physical containment (e.g. a biosafety cabinet, or BSC) or working at a higher biosafety level (BSL); using additional PPE; or completing an internal biosafety risk assessment.
    - This strategy is not necessary
    - This strategy doesn't seem necessary, but the team could consider it
    - The team should seriously consider this strategy
    - The team should not continue the project without this strategy

- Should the team implement additional biosecurity measures? This might include: increased security of the physical lab space or materials (e.g. adding access controls); increased security for computers or digital information; or more careful review of the people involved with the project.
    - This strategy is not necessary
    - This strategy doesn't seem necessary, but the team could consider it
    - The team should seriously consider this strategy
    - The team should not continue the project without this strategy

- Should the team evaluate the efficacy of existing countermeasures against the organisms or parts produced by the project? If no effective countermeasures (medical, detection, or otherwise) exist, the team would consult with iGEM about how to proceed.
    - This strategy is not necessary
    - This strategy doesn't seem necessary, but the team could consider it
    - The team should seriously consider this strategy
    - The team should not continue the project without this strategy

- Should the team craft a plan for responsible communication of the research results and review it with iGEM before proceeding? This might include: redacting specific information in light of security concerns; highlighting the biosafety and biosecurity

measures used during the course of the project; or emphasizing the public health or other beneficial uses of the project.
  - This strategy is not necessary
  - This strategy doesn't seem necessary, but the team could consider it
  - The team should seriously consider this strategy
  - The team should not continue the project without this strategy

- Should the team modify its experimental design or methodology? This might include: utilizing an attenuated strain or employing molecular containment measures that limit a strain's ability to proliferate outside the lab or within different hosts.
  - This strategy is not necessary
  - This strategy doesn't seem necessary, but the team could consider it
  - The team should seriously consider this strategy
  - The team should not continue the project without this strategy

- Should the team stop the project? It may be the case that no other options can sufficiently address the risks of the project. In this case, the team should stop the project.
  - This strategy is not necessary
  - This strategy doesn't seem necessary, but the team could consider it
  - The team should seriously consider this strategy
  - The team should not continue the project without this strategy

Please summarize your recommended risk management strategies. This might include specific training you've requested, additional biosafety and biosecurity measures, and/or modifications to experiment design or methodology. [open response]

5.11 Final background questions

As you completed this project review, did you do any additional research or consult any external resources (for example, by searching the web or asking personal contacts for their opinions)?

- Yes
- No
- If yes, roughly how much time did you spend doing additional research? [open response]

Is there anything else about this project review that you would like us to know? [open response]
